# Supplementary material for: The global diversity of Haemonchus contortus is shaped by human intervention and climate
Source: Nat Commun. 2019 Oct 22;10:4811. doi: 10.1038/s41467-019-12695-4 (PMC6805936; doi:10.1038/s41467-019-12695-4)
Supplement: Supplementary file 1 — Supplementary Information [file 41467_2019_12695_MOESM1_ESM.pdf]

The global diversity of *Haemonchus contortus* is shaped by human intervention and climate

Sallé et al.

Supplementary Information

Supplementary Tables **4**

Supplementary Table 1. Average nucleotide diversity by population 4

Supplementary Table 2. Population haplotype frequency at SNP in codon 167, 198, 200 of the *Hco-tbb-iso-1* and associated fenbendazole efficacy 1

Supplementary Table 3. Genotype counts at each codon position of *Hco-tbb-iso-1* according to the considered genotype likelihood (GL) cut-off 2

Supplementary Table 4. Significant GO term enrichment from genes under significant diversifying selection across pairwise comparisons 3

Supplementary Table 5. Bioclimatic variables definitions and codes 4

Supplementary Table 6. Significant associations between SNP markers and temperature annual range (BIO7) and annual precipitation (BIO12) bioclimatic variables 4

Supplementary Table 6 continued. 5

Supplementary Figures **6**

Supplementary Figure 1. Observed levels of nucleotide diversity in populations from France, Guadeloupe, and Namibia. 6

Supplementary Figure 2. Neighbour-joining tree inferred from the pairwise divergence between individual males 7

Supplementary Figure 3. Population clustering by means of a PCA applied to mitochondrial fixed variants derived from consensus sequences 8

Supplementary Figure 4. Pairwise FST estimates binned by MAF between populations with at least 5 individuals 9

Supplementary Figure 5. Admixture median absolute deviation for K clusters ranging from 2 to 10. 10

Supplementary Figure 6. Admixture pattern across populations for K values of 2, and 4 to 10. 11

Supplementary Figure 7. Bayesian coalescent-based consensus tree of mitochondrial genomes 12

Supplementary Figure 8. Tajima's *D* estimate plotted against genomic position. 13

Supplementary Figure 9. Reduction of genetic diversity in the vicinity of *Hco-tbb-iso-1* locus for three benzimidazole-resistant and two benzimidazole-susceptible populations. 14

Supplementary Figure 10. Phylogenetic network based on pairwise allelic divergence at SNP loci spanning the *Hco-tbb-iso-1* locus. 15

Supplementary Figure 11. Topology weighing analysis of a 100-Kbp window centred on *Hco-tbb-iso-1*. 16

Supplementary Figure 12. Mean coverage of genotypic group at SNP in codon positions 167, 198 and 200 of *Hco-btub-1.* 17

Supplementary Figure 13. Individual genotypes at mutant SNP positions of *Hco-tbb-iso1* inferred from genotype likelihoods. 18
Supplementary Figure 14. XP-CLR selection score plotted against genomic position. 19
Supplementary Figure 15. Differentiation signal between populations from temperate, tropical and arid environments 20
Supplementary Figure 16. Pairwise Pearson's correlations (a) and principal component analysis (b) between environmental variables from eight populations 22
Supplementary Figure 17. Variant Quality Score Recalibration (VSQR) summary statistics 23
Supplementary Methods **24**
Creation of a reference "truth" SNP database 24
Effective population size estimation with MSMC2 24
Modeling population demographics and divergence dating 25
Phylogenetic analysis of mitochondrial coding sequences 26
Supplementary Notes **28**
Supplementary Note 1. Evaluation of genotype analysis frameworks in face of low coverage samples 28
Supplementary Figure 18. Coverage improvement for a subset of 43 individual *Haemonchus contortus* males 28
Supplementary Figure 19. The relationship between sample coverage and population diversity estimates 29
Supplementary Figure 20. The relationship between Tajima's *D* estimates and coverage 30
Supplementary Figure 21. The relationship between pairwise F_{ST} estimates and coverage according to the considered framework 31
Supplementary Figure 22. The relationship between Hamming's distance and coverage 32
Supplementary Figure 23. Principal component analysis based on nuclear VQSR SNP calls across 223 individuals 33
Supplementary Figure 24. Admixture analysis run for chromosome II on the same set of 43 individuals before (left) or after (right) resequencing for K ranging from 2 to 5 34
Supplementary Note 2. Beagle imputation accuracy 35
Supplementary Figure 25. Genotype discordance after Beagle imputation 36
Supplementary references **37**

Supplementary Tables

Supplementary Table 1. Average nucleotide diversity by population

Population	Mean π across nuclear genome	Standard deviation	Population mean coverage
ACO	0.00681	0.00096	1.79
AUS.1	0.00613	0.00044	2.57
AUS.2	0.00509	0.00032	2.74
BEN	0.00939	0.00232	0.33
CAP	0.00721	0.00104	0.70
FRA.1	0.00723	0.00037	3.32
FRA.2	0.00544	0.00059	1.82
FRA.3	0.00623	0.00084	0.48
GUA	0.01078	0.00032	4.40
IND	0.00555	0.00075	4.95
MOR	0.00820	0.00044	3.50
NAM	0.01300	0.00075	5.62
STA.1	0.00669	0.00183	4.01
STA.2	0.00442	0.00045	1.79
STA.3	0.01124	0.00090	2.64
STO	0.00975	0.00075	2.61
ZAI	0.00858	0.00029	2.11
FRA.1*	0.00610	0.00049	8.03
GUA*	0.01063	0.00079	12.75
NAM*	0.01136	0.00079	9.85

Asterisks indicates population subset of 5 individuals with minimum mean coverage of 5×: this was limited to France (FRA.1, n = 5, mean coverage of 7.66×), Guadeloupe (GUA, n = 5, mean coverage of 12.75×) and Namibia (NAM, n = 6, mean coverage of 9.85×).

Supplementary Table 2. Population haplotype frequency at SNP in codon 167, 198, 200 of the *Hco-tbb-iso-1* and associated fenbendazole efficacy

.

Population	A/A-A/A-T/T	A/T-A/A-T/T	T/T-A/A-A/A	T/T-A/A-T/A	**T/T-A/A-T/T**	T/T-C/A-A/T	T/T-C/C-T/T	Fenbendazole efficacy
ACO	0	0	0	0	1	0	0	N/A
AUS.1	0	0	5	0	0	1	2	43
AUS.2	0	0	0	0	4	0	0	100
BRA	0	0	1	0	0	0	0	0.795 μg/ml[a]
CAP	0	0	0	0	1	0	0	N/A
FRA.1 - Farm 1	0	0	2	0	0	0	0	60.2
FRA.1 - Farm 2	0	0	0	0	0	0	4	64.3
FRA.1 - Farm 3	0	0	2	0	0	0	0	N/A
FRA.1 - Farm 4	0	0	1	0	0	1	0	50.4
FRA.1 - Farm 5	1	1	0	0	0	0	0	98.6
FRA.1 - Farm 6	0	0	0	0	0	0	2	60.4
FRA.1 - Farm 8	0	0	2	0	0	0	0	43.7
FRA.2	0	0	0	0	3	0	0	100
FRA.4	0	0	0	0	1	0	0	100
GUA	0	0	14	0	0	0	0	N/A
IND	0	0	0	0	1	0	0	N/A
MOR	0	0	1	0	1	0	0	N/A
NAM	0	0	4	1	0	0	0	N/A
STA.1	0	0	1	0	1	1	2	0.587 μg/ml[a]
STA.2	0	0	0	0	2	0	0	N/A
STA.3	0	0	6	0	0	0	0	33.75[b]
STO	0	0	0	0	3	0	0	N/A
ZAI	0	0	0	0	1	0	0	100

For each population, the total count of each genotype combination at codon positions 167/198/200 is listed (based on individuals showing minimum genotype likelihood of 60%). Fully susceptible reference genotype is T/T-A/A-T/T for codon positions 167 -198 - 200. Any variation in this sequence indicates the recessive mutant. Unless stated otherwise, benzimidazole efficacy refers to Faecal Egg Count Reduction Test (FECRT, in %) value after fenbendazole treatment.

a: Median inhibitory concentration (IC50) for triclabendazole measured by means of an egg hatch assay and relative to a susceptible isolate showing an IC50 of 0.022 μg/ml.

b: FECRT value after albendazole treatment[1].

Supplementary Table 3. Genotype counts at each codon position of *Hco-tbb-iso-1* according to the considered genotype likelihood (GL) cut-off

	P167Y			E198A			P200Y		
	A/A	T/A	**T/T**	**A/A**	A/C	C/C	A/A	T/A	**T/T**
GL > 0	1	1	**221**	**208**	3	12	55	5	**166**
GL > 0.6	1	1	**72**	**61**	3	10	39	4	**31**
GL > 0.8	0	0	**31**	**25**	3	3	17	4	**10**

Reference genotypes are indicated in bold.

Genotypes for the *Hco-tbb-iso-1* locus were determined for each of the 223 samples using ANGSD[2]. Analysis of all genotypes prior to filtering (GQ > 0) revealed an over-representation of susceptible genotypes at each position (Supplementary Table 3).

A regression analysis of genotype counts at position 200 upon sample mean coverage demonstrated a significant bias ($P = 6.64 \times 10^{-8}$, $F_{2,220} = 17.83$) toward reference susceptible genotype T/T in samples with lower coverage (-0.8x for this particular subpopulation). The same applied for position 198 (Kruskal-Wallis $\chi^2 = 35.423$, $df = 2$, $P = 0.05$).

This bias was corrected for by selecting the only genotype with GL above 60%, resulting in 0.04x difference between homozygous genotypes (Kruskal-Wallis $\chi^2 = 3.11$, $df = 2$, $P = 0.2115$ and $\chi^2 = 3.74$, $df = 2$, $P = 0.15$ for the P200Y and E198A positions respectively; Supplementary Fig. 13). Samples with the reference genotype at codon position 200, still displayed a lower coverage than their counterparts. Applying a more stringent GL cut-off resulted in the same pattern, ruling out a coverage bias as population coverage reached an average of 6x [2 – 15.24] in this case. Genotype prediction corroborated known fenbendazole resistance status of Australian (AUS.1) and South-African (STA.3) populations (Supplementary Fig. 13) and were also in line for the French population despite the low number of observations available (Supplementary Table 2).

Supplementary Table 4. Significant GO term enrichment from genes under significant diversifying selection across pairwise comparisons

GO term	GO term description	*P* value
GO:0004674	protein serine/threonine kinase activity	1.30E-04
GO:0005326	neurotransmitter transporter activity	5.80E-04
GO:0006448	regulation of translational elongation	6.00E-04
GO:0016746	transferase activity, transferring acyl groups	1.17E-03
GO:0043412	macromolecule modification	2.40E-03
GO:0060249	anatomical structure homeostasis	3.10E-03
GO:0044428	nuclear part	3.60E-03
GO:0009898	cytoplasmic side of plasma membrane	3.80E-03
GO:0051539	4 iron, 4 sulfur cluster binding	4.14E-03
GO:0042493	response to drug	4.30E-03
GO:0000226	microtubule cytoskeleton organization	4.60E-03
GO:0016627	oxidoreductase activity, acting on the CH-CH group of donors	5.40E-03
GO:0001714	endodermal cell fate specification	5.50E-03
GO:0043051	regulation of pharyngeal pumping	5.80E-03
GO:0006950	response to stress	5.90E-03
GO:0010628	positive regulation of gene expression	6.80E-03
GO:0002181	cytoplasmic translation	7.10E-03
GO:0048029	monosaccharide binding	7.13E-03
GO:0016634	oxidoreductase activity, acting on the CH-CH group of donors, oxygen as acceptor	7.63E-03
GO:0071949	FAD binding	7.63E-03
GO:0030119	AP-type membrane coat adaptor complex	7.90E-03
GO:0046662	regulation of oviposition	8.60E-03
GO:0015299	solute:proton antiporter activity	8.66E-03
GO:0016757	transferase activity, transferring glycosyl groups	8.88E-03
GO:0001505	regulation of neurotransmitter levels	9.10E-03
GO:0006417	regulation of translation	9.40E-03

Supplementary Table 5. Bioclimatic variables definitions and codes

Code	Bioclimatic variable
BIO1	Annual Mean Temperature
BIO2	Mean Diurnal Range (Mean of monthly (max temp - min temp))
BIO3	Isothermality (BIO2 / BIO7)* 100
BIO4	Temperature Seasonality (standard deviation *100)
BIO5	Max Temperature of Warmest Month
BIO6	Min Temperature of Coldest Month
BIO7	Temperature Annual Range (BIO5 - BIO6)
BIO8	Mean Temperature of Wettest Quarter
BIO9	Mean Temperature of Driest Quarter
BIO10	Mean Temperature of Warmest Quarter
BIO11	Mean Temperature of Coldest Quarter
BIO12	Annual Precipitation
BIO13	Precipitation of Wettest Month
BIO14	Precipitation of Driest Month
BIO15	Precipitation Seasonality (Coefficient of Variation)
BIO16	Precipitation of Wettest Quarter
BIO17	Precipitation of Driest Quarter
BIO18	Precipitation of Warmest Quarter
BIO19	Precipitation of Coldest Quarter

Supplementary Table 6. Significant associations between SNP markers and temperature annual range (BIO7) and annual precipitation (BIO12) bioclimatic variables

Variable	Chromosome	Position	Adjusted P-value	Underlying gene	*C. elegans* ortholog
BIO12	I	4417279	4.03642E-06	*HCOI01130100*	-
BIO12	I	4436114	6.02015E-07	-	-
BIO12	I	7177538	4.73872E-05	-	-
BIO12	I	7178096	5.30283E-06	-	-
BIO12	I	7178137	1.38924E-06	-	-
BIO12	I	7178161	3.33913E-06	-	-
BIO12	I	7178216	2.11838E-06	-	-
BIO12	I	7183000	1.28655E-05	-	-
BIO12	I	7183089	3.08945E-05	-	-
BIO12	I	7183617	4.44307E-06	-	-
BIO12	I	25362173	5.21215E-05	*HCOI02027500*	*nlp-40*

Supplementary Table 6 continued.

Variable	Chromosome	Position	Adjusted P-value	Underlying gene	*C. elegans* ortholog
BIO12	I	25362301	2.08639E-05	*HCOI02027500*	*nlp-40*
BIO12	I	40725432	1.17771E-05	*HCOI01509500*	*Y37E3.1*
BIO12	II	17957	1.34973E-06	*HCOI00712600*	-
BIO12	II	2007776	3.54502E-05	-	-
BIO12	II	8956597	7.79071E-06	*HCOI01769300*	-
BIO12	II	19715744	4.35577E-06	-	-
BIO12	II	19715795	2.83746E-05	-	-
BIO12	II	19715876	8.48139E-07	-	-
BIO12	II	33562640	3.59797E-05	-	-
BIO12	II	33562649	1.9803E-05	-	-
BIO12	II	33563060	3.22084E-05	-	-
BIO12	III	13983070	1.17951E-06	-	-
BIO12	III	13983103	1.33359E-05	-	-
BIO12	III	13988815	6.29649E-06	-	-
BIO12	III	37491795	2.12507E-06	-	-
BIO12	III	37492340	2.99508E-05	-	-
BIO7	III	26823004	1.6131E-05	*HCOI00198200*	*anp-1*
BIO7	III	26823010	2.52474E-06	*HCOI00198200*	*anp-1*
BIO7	III	26824172	7.26791E-06	*HCOI00198200*	*anp-1*
BIO7	III	26824197	1.38644E-07	*HCOI00198200*	*anp-1*
BIO7	III	26824240	2.26566E-09	*HCOI00198200*	*anp-1*
BIO7	III	34817063	4.81281E-06	-	-
BIO7	IV	15905141	2.51251E-05	-	-
BIO7	IV	15905191	8.86985E-07	-	-
BIO7	IV	16544889	3.10134E-05	*HCOI00312500*	*C53B4.1, Y57G11C.23, ZK892.3*
BIO7	IV	21127120	3.46281E-06	-	-
BIO7	IV	24262808	1.43673E-05	-	-
BIO7	IV	30389515	3.68264E-06	-	-
BIO7	IV	36652651	2.83202E-05	*HCOI02015300*	*ugt-45*
BIO7	IV	51765259	2.84043E-05	*HCOI01461000*	*Y5F2A.4*
BIO7	V	41114841	3.90813E-06	*HCOI00078600*	-

Supplementary Figures

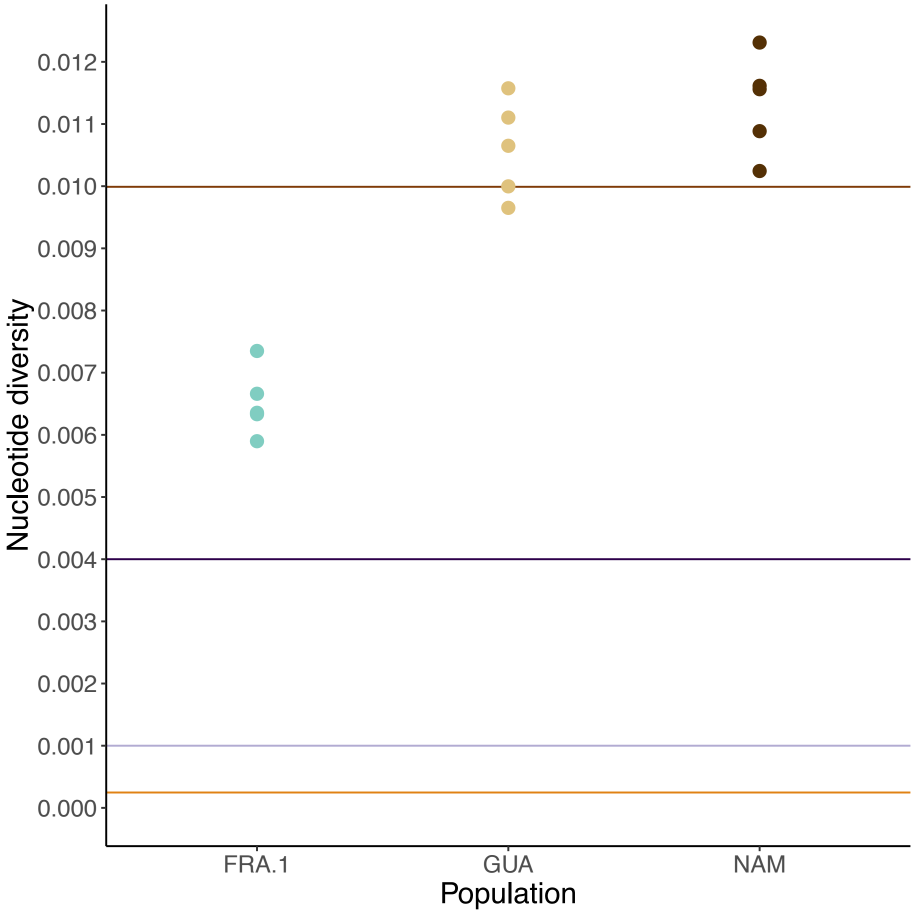

Supplementary Figure 1. Observed levels of nucleotide diversity in populations from France, Guadeloupe, and Namibia.

Nucleotide diversity (π) is presented representing the number of substitutions per base for three subsets of populations built from individuals with a mean coverage of 5x and more, i.e. France (FRA.1, n = 5, mean coverage of 7.66×), Guadeloupe (GUA, n = 5, mean coverage of 12.75×) and Namibia (NAM, n = 6, mean coverage of 9.85×). Each dot corresponds to the average nucleotide diversity across single autosome. Horizontal lines provide genome-wide nucleotide diversity estimates from two other parasitic nematodes (Onchocercidae, clade III): *Wuchereria bancrofti*[3] ($\pi = 2.7 \times 10^{-4}$, orange), and *Onchocerca volvulus*[4] ($\pi_S = 4 \times 10^{-3}$ and $\pi_N = 1 \times 10^{-3}$ in dark purple and light purple respectively). Brown horizontal line matches previously reported values for *Drosophila melanogaster*[5] (0.999% averaged across three populations showing values of 0.00531, 0.00752, 0.01714).

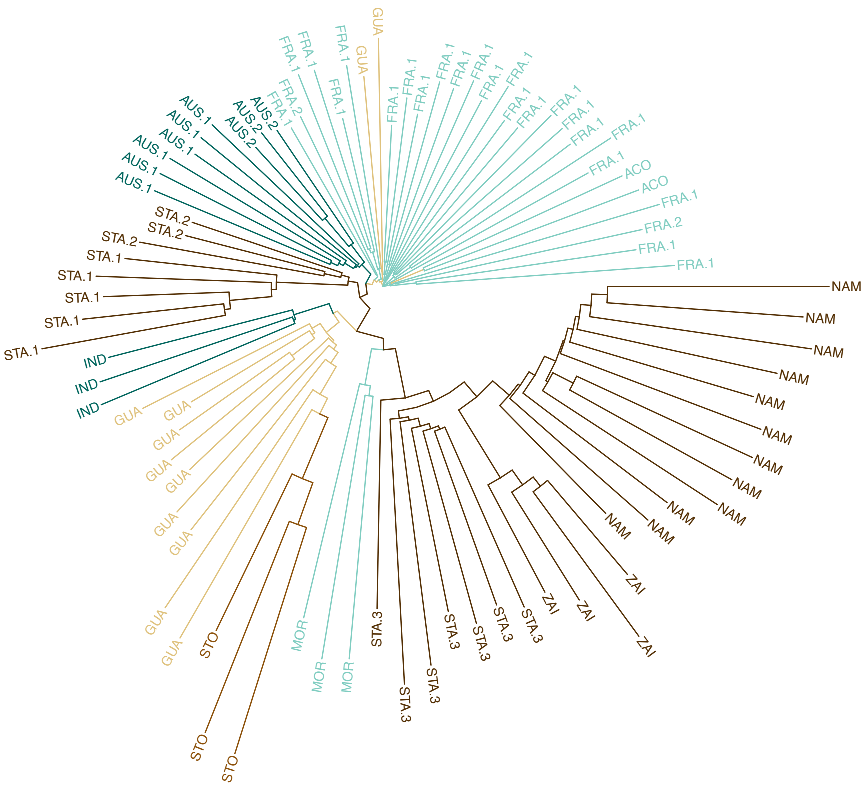

Supplementary Figure 2. Neighbour-joining tree inferred from the pairwise divergence between individual males

Pair-wise Hamming's distance, *i.e.* the SNP fraction non-identical-by-state between two individuals, were computed with PLINK[6] v1.90b3v. Because of coverage bias, the analysis was restricted to 75 individuals with a minimum mean coverage of 2.5x. Colours correspond to the samples geographical origins (sand: South-America, brown: Western-Africa, light green: Mediterranean area, dark brown: Subtropical Africa, dark green: Australia).

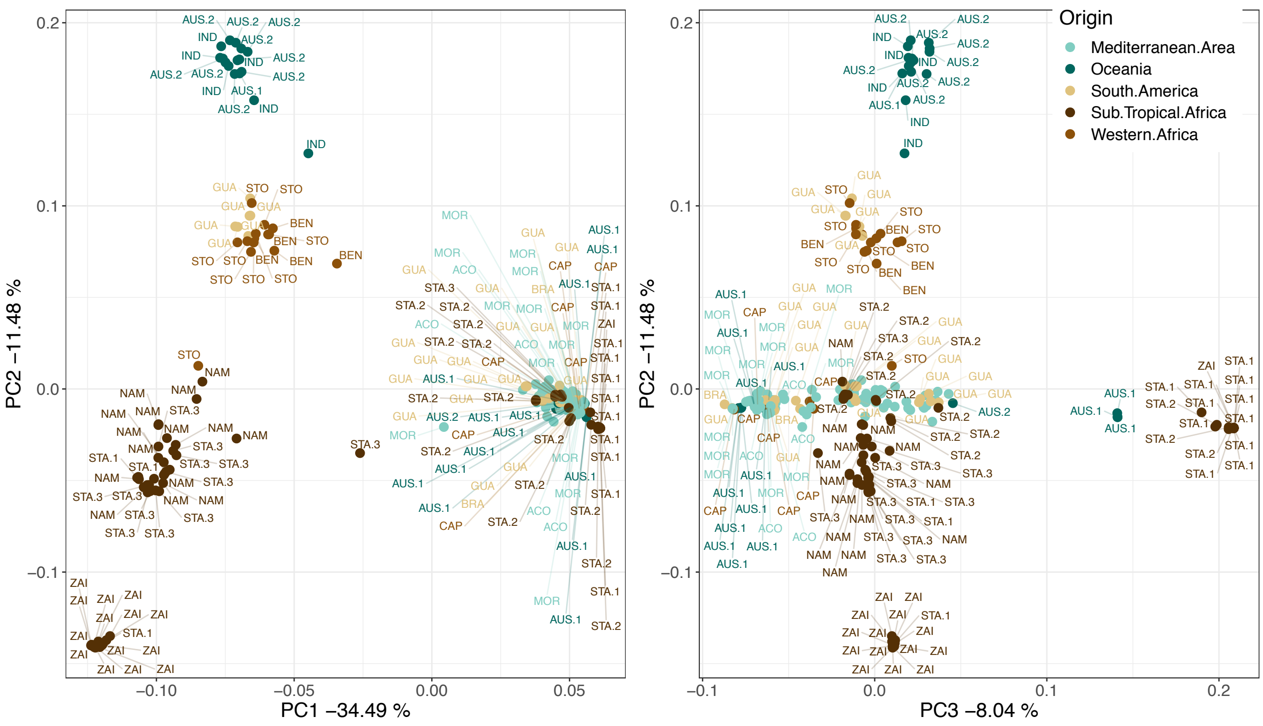

Supplementary Figure 3. Population clustering by means of a PCA applied to mitochondrial fixed variants derived from consensus sequences

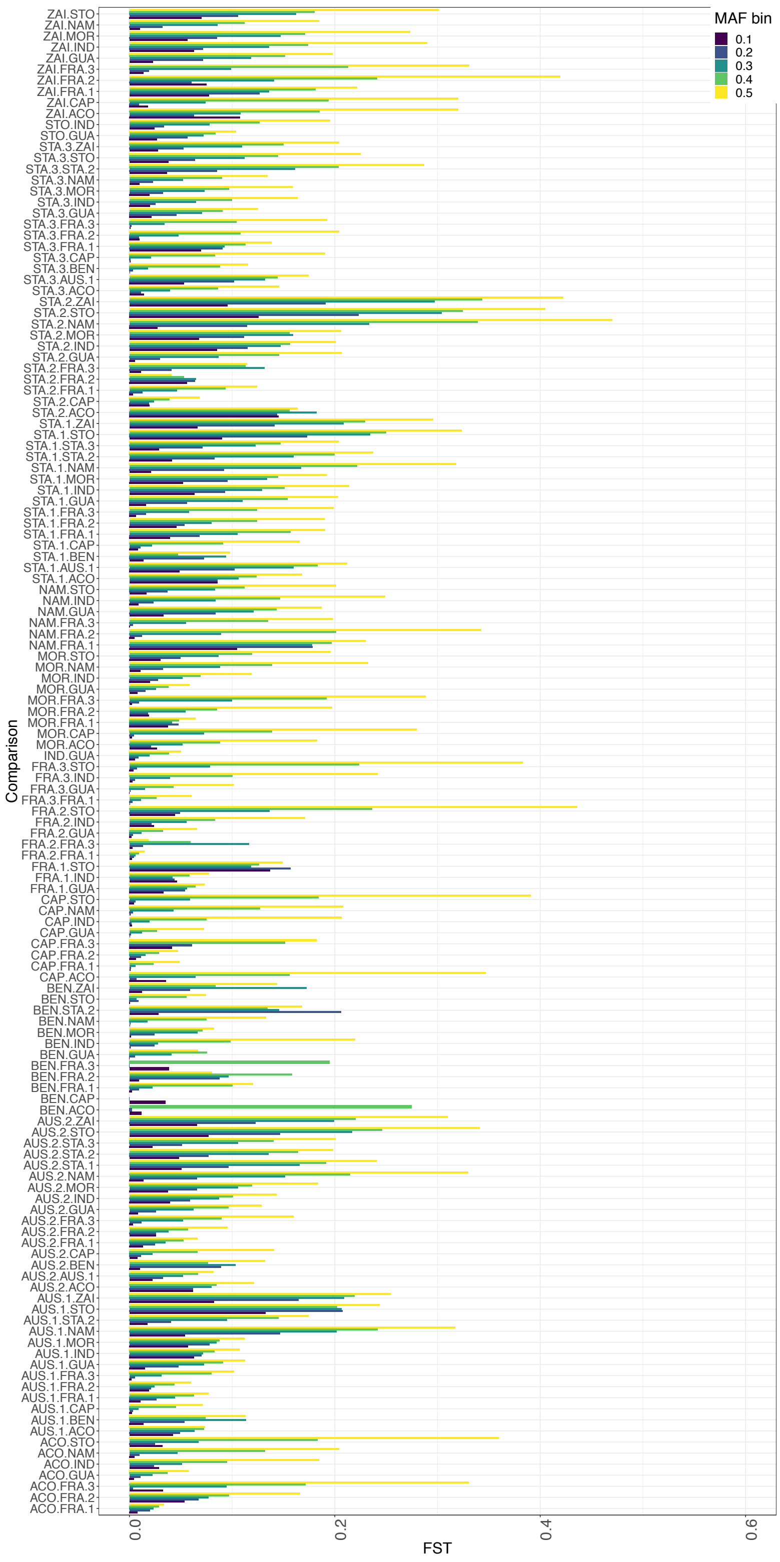

Supplementary Figure 4. Pairwise FST estimates binned by MAF between populations with at least 5 individuals

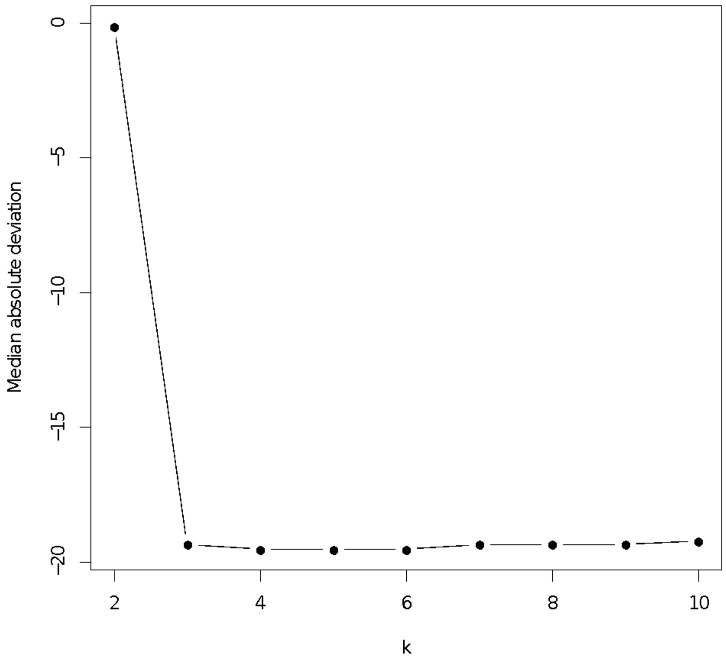

Supplementary Figure 5. Admixture median absolute deviation for K clusters ranging from 2 to 10.

Median absolute deviation was estimated across five runs of NGSAdmix[7], retaining sites with less than 50% missing data across individuals and minor allele frequency (MAF) above 5% and leaving one autosome out at a time.

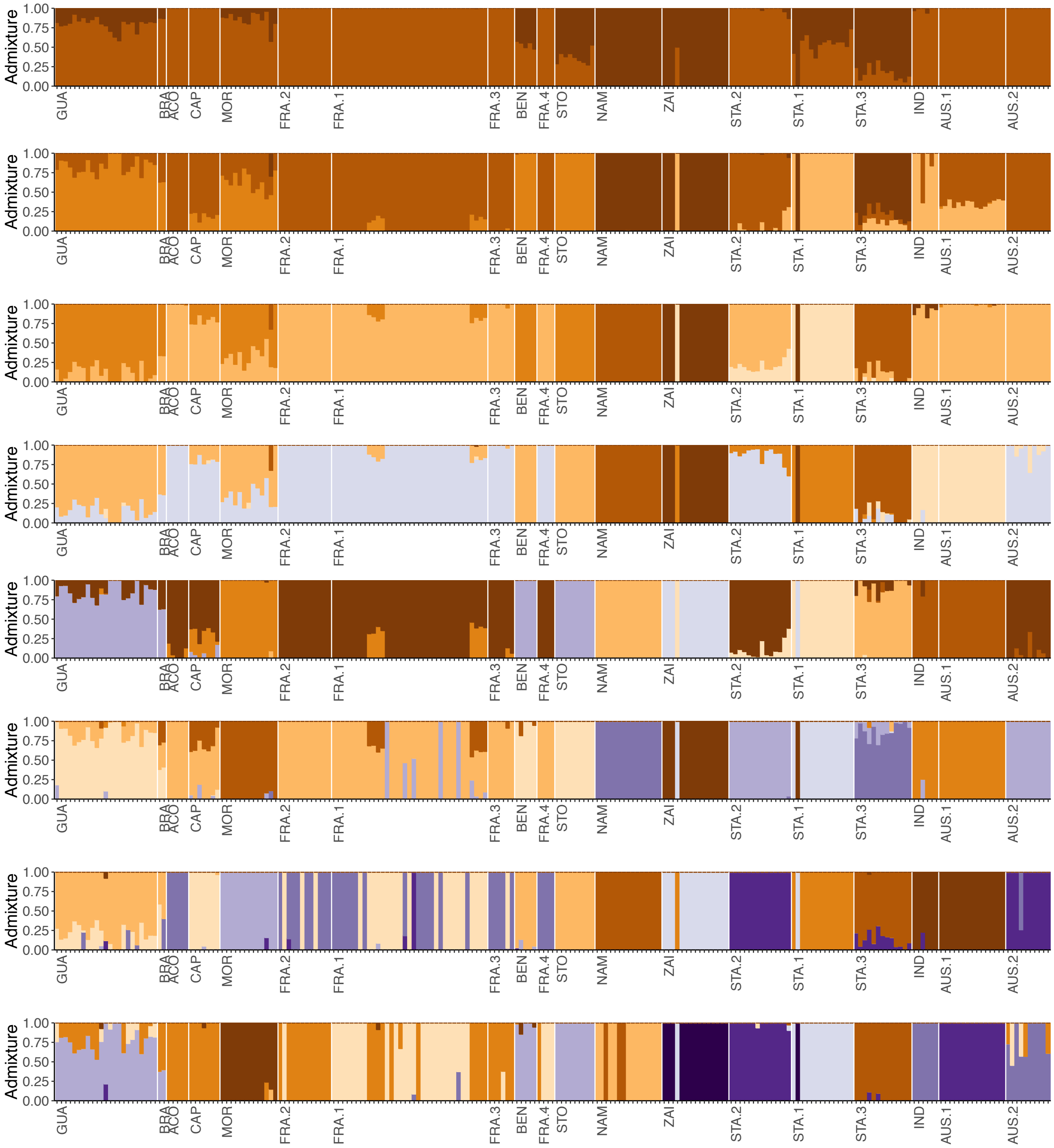

Supplementary Figure 6. Admixture pattern across populations for K values of 2, and 4 to 10.

Admixture pattern (determined from sites with minor allele frequency above 5% and call rate higher than 50%) is represented for the 223 considered individuals, sorted along their longitudinal range. Cluster size of K = 2 is presented on top down to K = 10, each color corresponding to one cluster.

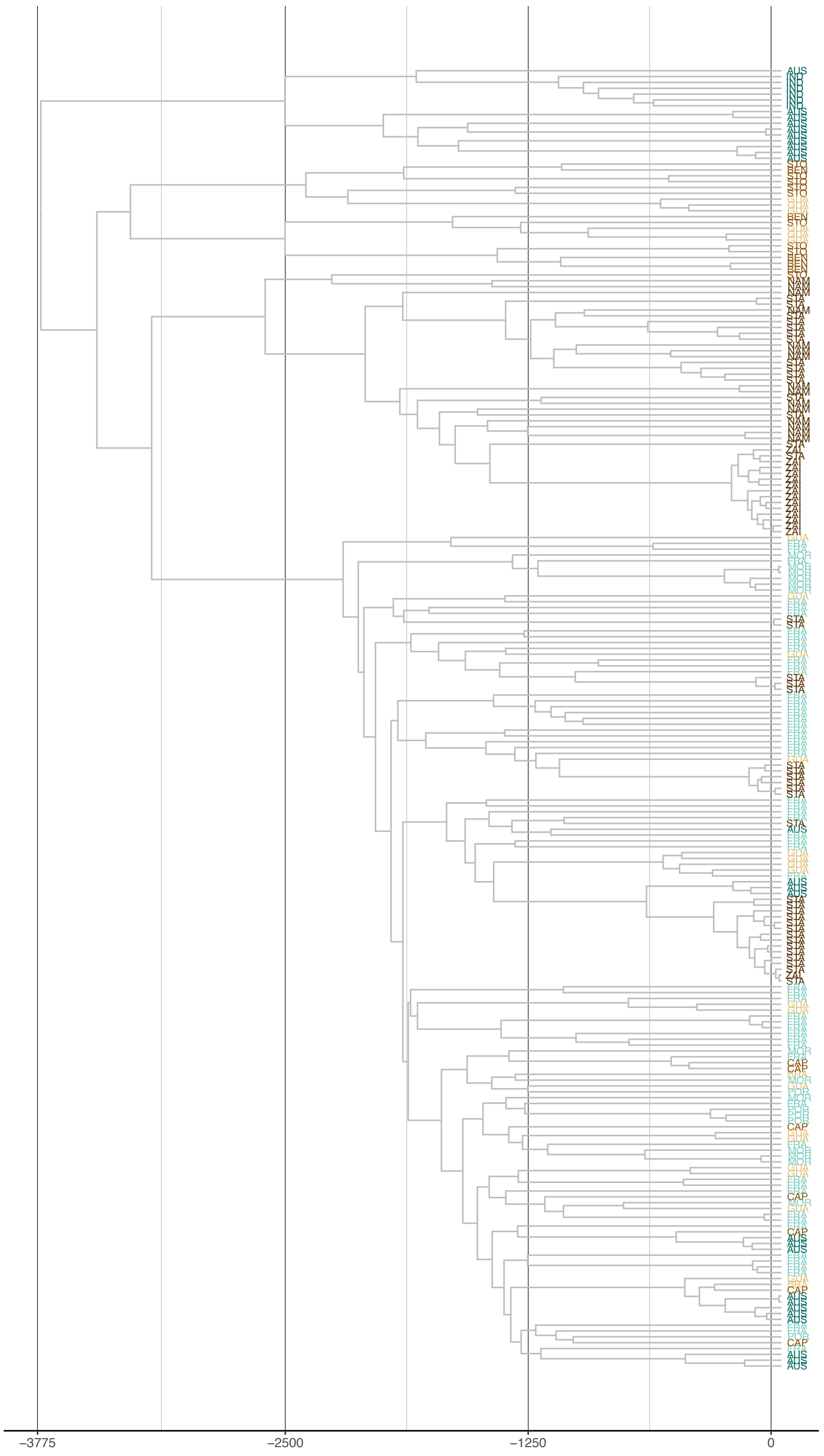

Supplementary Figure 7. Bayesian coalescent-based consensus tree of mitochondrial genomes

Maximum clade credibility tree is represented from 50 million Monte-Carlo Markov Chains, and burn-in of the first 20 million iterations. Tips are coloured according to the sample geographical location following the same key as in Fig.1a. Vertical lines correspond to 1250-year intervals.

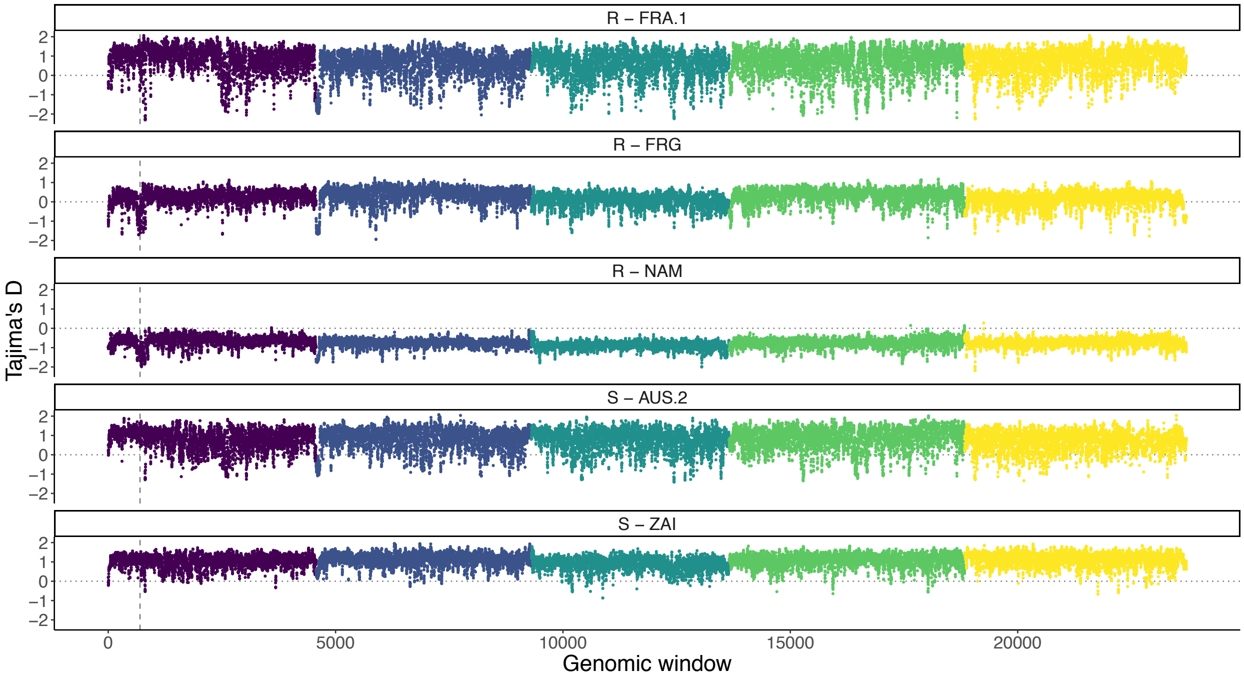

Supplementary Figure 8. Tajima's *D* estimate plotted against genomic position.

Plot represents Tajima's *D* coefficient along the genome (from chromosome I in purple to V in yellow) for five populations with the highest representation of individuals with a mean depth of coverage higher than 5x. Vertical dash line indicates β-tubulin locus.

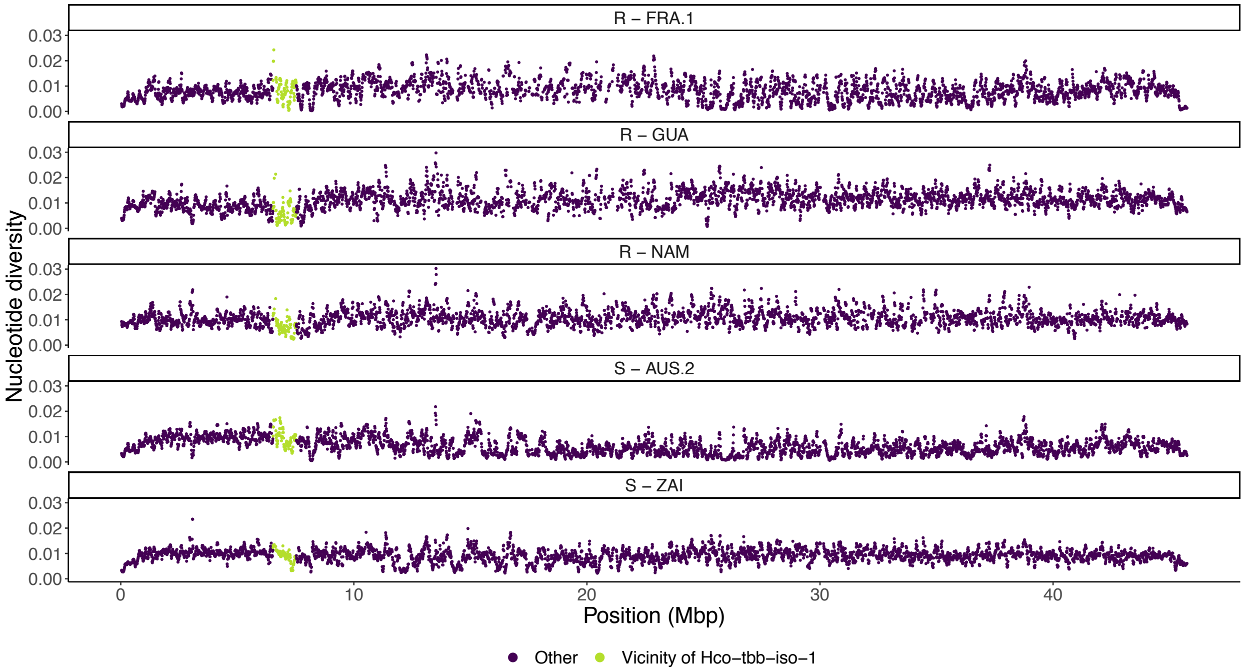

Supplementary Figure 9. Reduction of genetic diversity in the vicinity of *Hco-tbb-iso-1* locus for three benzimidazole-resistant and two benzimidazole-susceptible populations.

Plot represents nucleotide diversity along chromosome I for five populations with the highest representation of individuals with a mean depth of coverage higher than 5x (R: resistant; S: susceptible). Green dots highlight the diversity reduction within a 1-Mbp window centred at the *β*-tubulin locus (mean π = 0.007 across resistant populations) relative to chromosome I (mean π = 0.011 across resistant populations, purple dots). Diversity in susceptible populations was slightly higher at the *β*-tubulin locus (mean π = 0.009 across resistant populations) relative to chromosome I (mean π = 0.008 across resistant populations). Direct comparison between resistant and susceptible populations was not possible because of the difference in mean coverage.

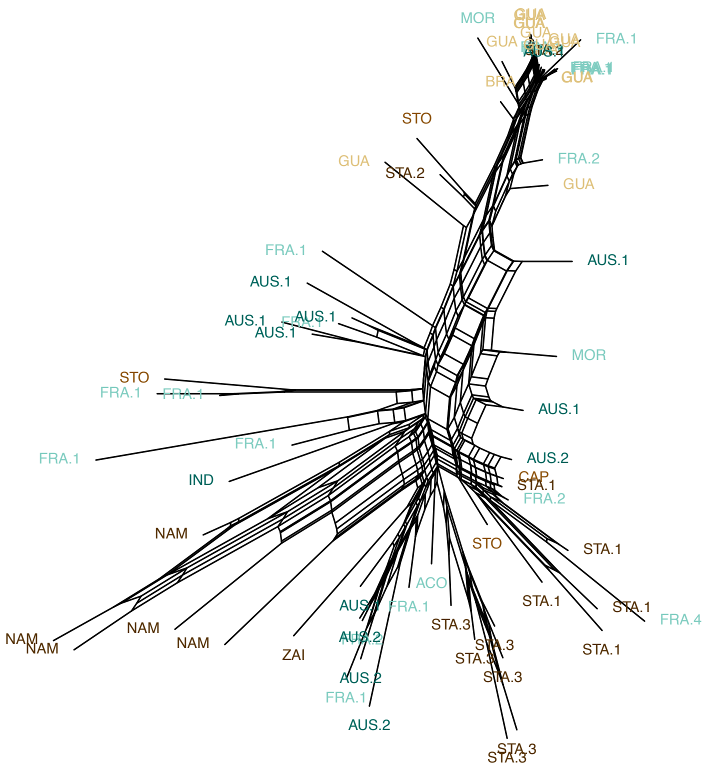

Supplementary Figure 10. Phylogenetic network based on pairwise allelic divergence at SNP loci spanning the *Hco-tbb-iso-1* locus.

Number of pairwise allelic differences between phased genotypes over the *Hco-tbb-iso1* locus (2,158 bp, 296 SNP positions considered) were counted for the 74 individuals with minimal genotype likelihood of 60%. The neighbour-net graph reveals resistant populations are largely polyphyletic, e.g. Namibia (NAM) or South-Africa (STA.3 and STA.1), whereas FRA.1 and GUA individuals tend to cluster in the same way. Isolates are coloured by geographical region (sand: South-America, brown: Western-Africa, light green: Mediterranean area, dark brown: Subtropical Africa, dark green: Australia).

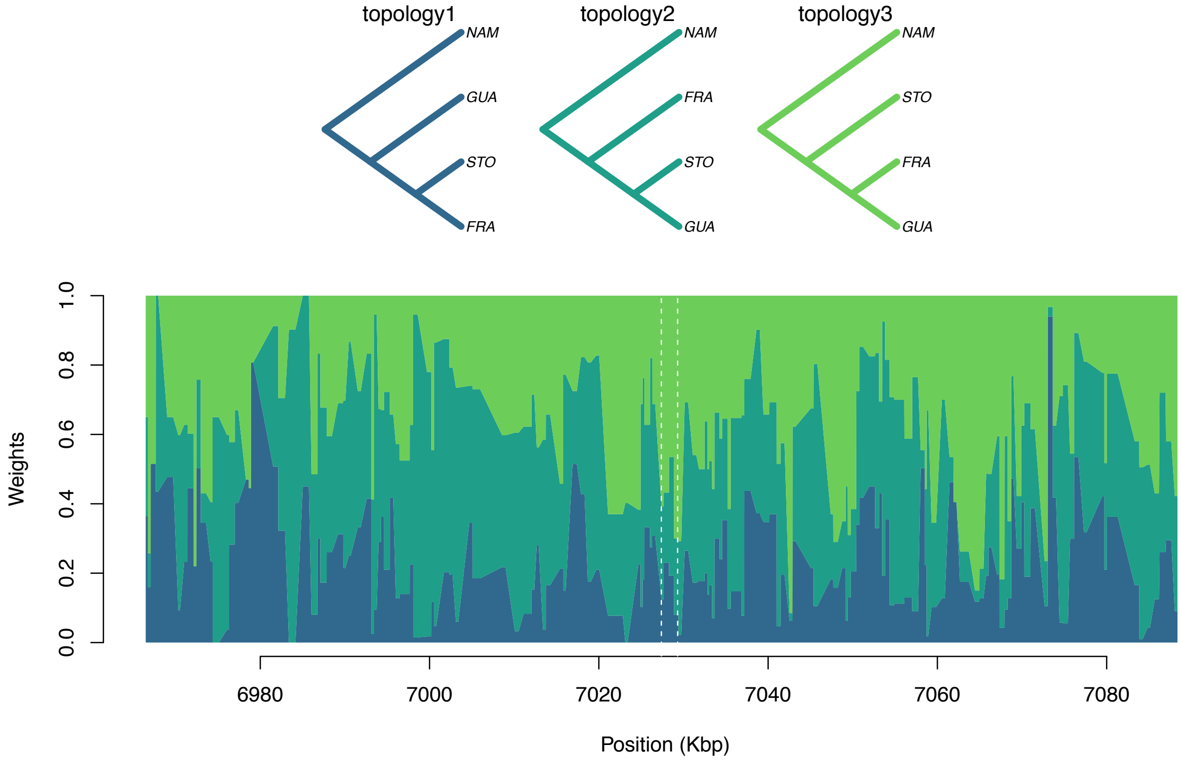

Supplementary Figure 11. Topology weighing analysis of a 100-Kbp window centred on *Hco-tbb-iso-1*.

Topology weighing analysis of a 20-Kbp window centred on *Hco-tbb-iso-1*, using populations from Namibia (NAM), France (FRA.1), Guadeloupe (GUA) and São Tomé (STO). At each position, the weight of each of the three possible topologies inferred from 50 Kbp-windows is overlaid. Topology 2 (dark green) corresponds to an isolation-by-distance history, while topology 3 (light green) would agree with shared genetic material between worm populations from French mainland into Guadeloupe. The position of *Hco-tbb-iso-1* locus is indicated by the vertical dashed lines. This figure supports the gene flow event and the analysis using Moroccan worms as a link population with GUA.

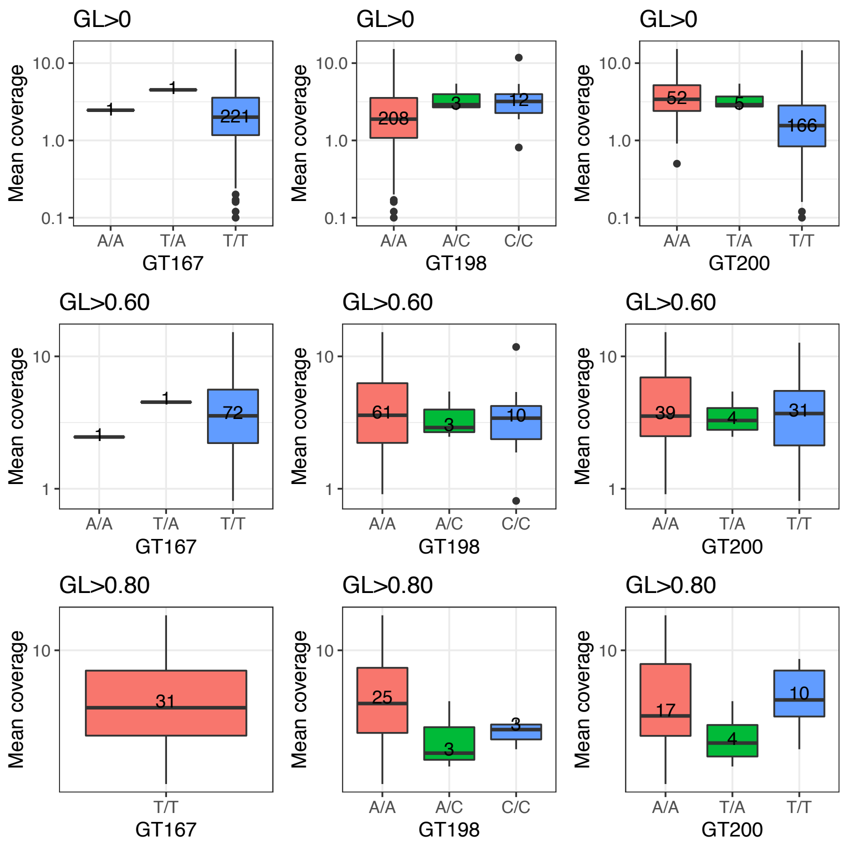

Supplementary Figure 12. Mean coverage of genotypic group at SNP in codon positions 167, 198 and 200 of *Hco-btub-1.*

For each codon position (GT167, 198 and 200) and genotype likelihood (GL) cut-off, genotypic group mean coverage is represented, showing a coverage bias in reference genotypic group when no GL filtering is applied. No significant impact on the relationship between predicted genotype and samples mean coverage is observed between GL cut-off of 0.6 or 0.8. Number of observations per group are indicated within each box.

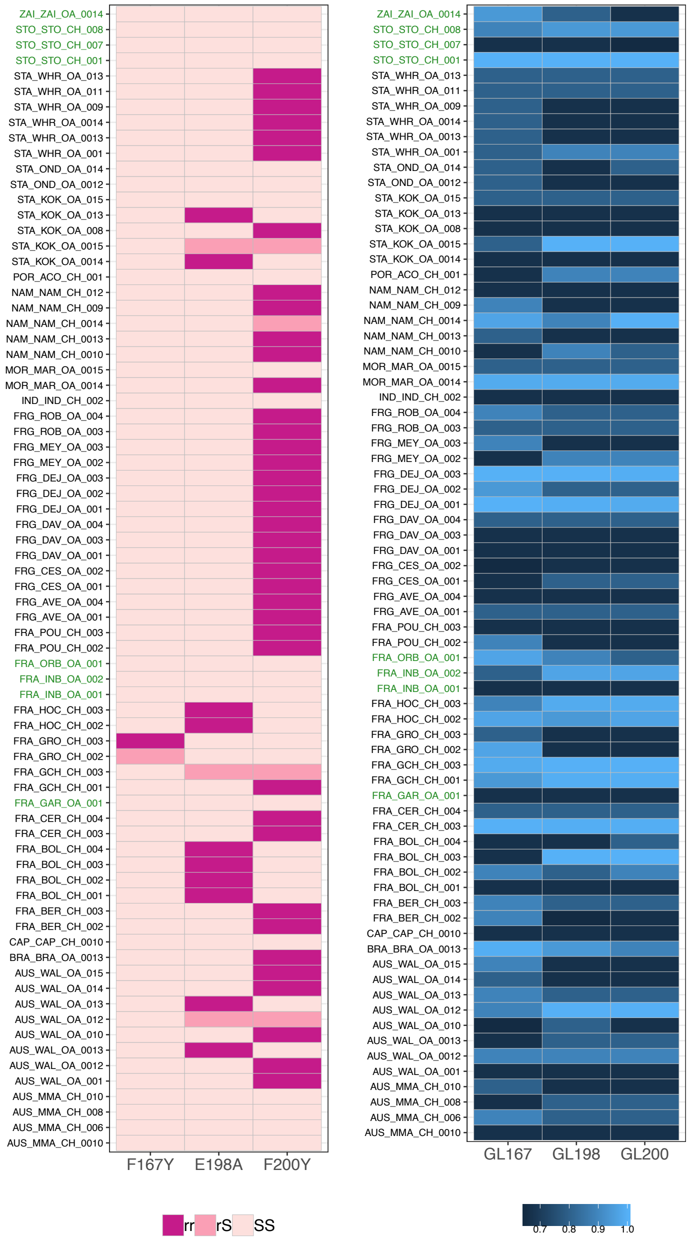

Supplementary Figure 13. Individual genotypes at mutant SNP positions of *Hco-tbb-iso1* inferred from genotype likelihoods.

The left panel displays the individual (row) combination of the genotypes at codon position 167, 198 and 200 (column) of the *Hco-tbb-iso-1* locus. Colour intensity follows the mutant allele dosage, i.e. purple standing for mutant homozygotes (rr), salmon for heterozygotes and pink shows homozygote reference genotype. Populations with phenotypically susceptible worms are coloured in green. The right panel represents the genotype likelihood of each individual-locus combination, ranging from 60% to 100% probability, and showing the absence of bias between coverage and genotype.

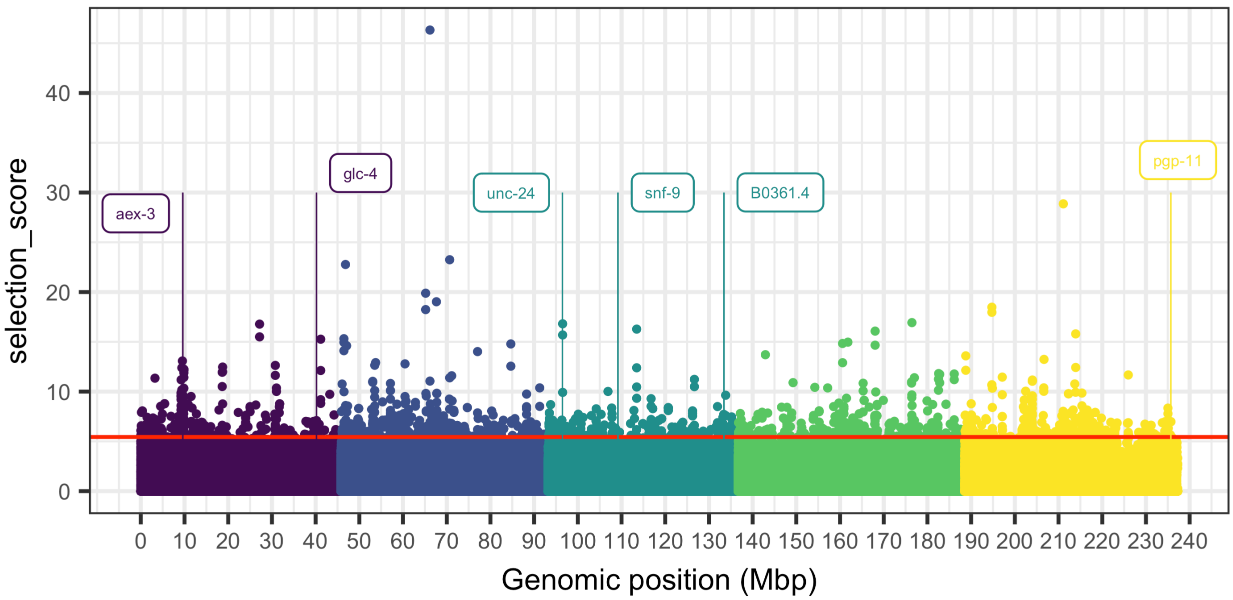

Supplementary Figure 14. XP-CLR selection score plotted against genomic position.

Each point represents the selection score generated by XP-CLR by its position in the genome. Positions are given in Mbp, and points are coloured by chromosome (from chromosome I in purple to chromosome V in yellow). The vertical grey dashed line indicates the *Hco-tbb-iso-1* locus, and the horizontal red line corresponds to the top 0.1% quantile. Coloured vertical lines and boxes point at candidate genes of interest, being either members of the *dauer* pathway (*daf-36, tax-4*), or putative candidates underpinning ivermectin resistance.

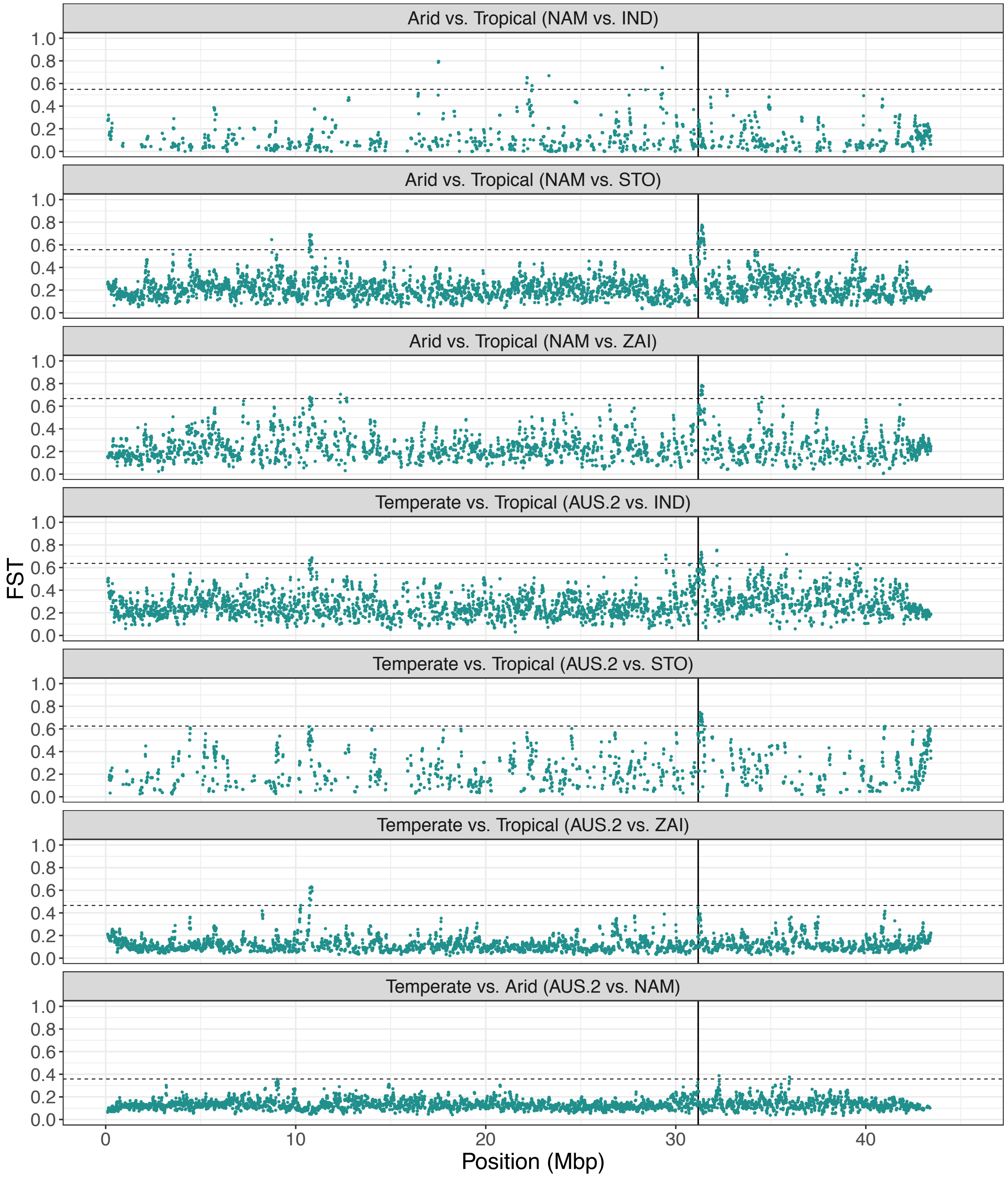

Supplementary Figure 15. Differentiation signal between populations from temperate, tropical and arid environments

This figure supports the strong genetic differentiation over the Polycomb protein coding gene ortholog (vertical line) between additional populations from Australia (AUS.2), Indonesia (IND), Namibia (NAM), São Tomé (STO) and Zaire (ZAI). The dashed horizontal line materializes the considered cut-off for significant differentiation (3 standard deviation from the mean).

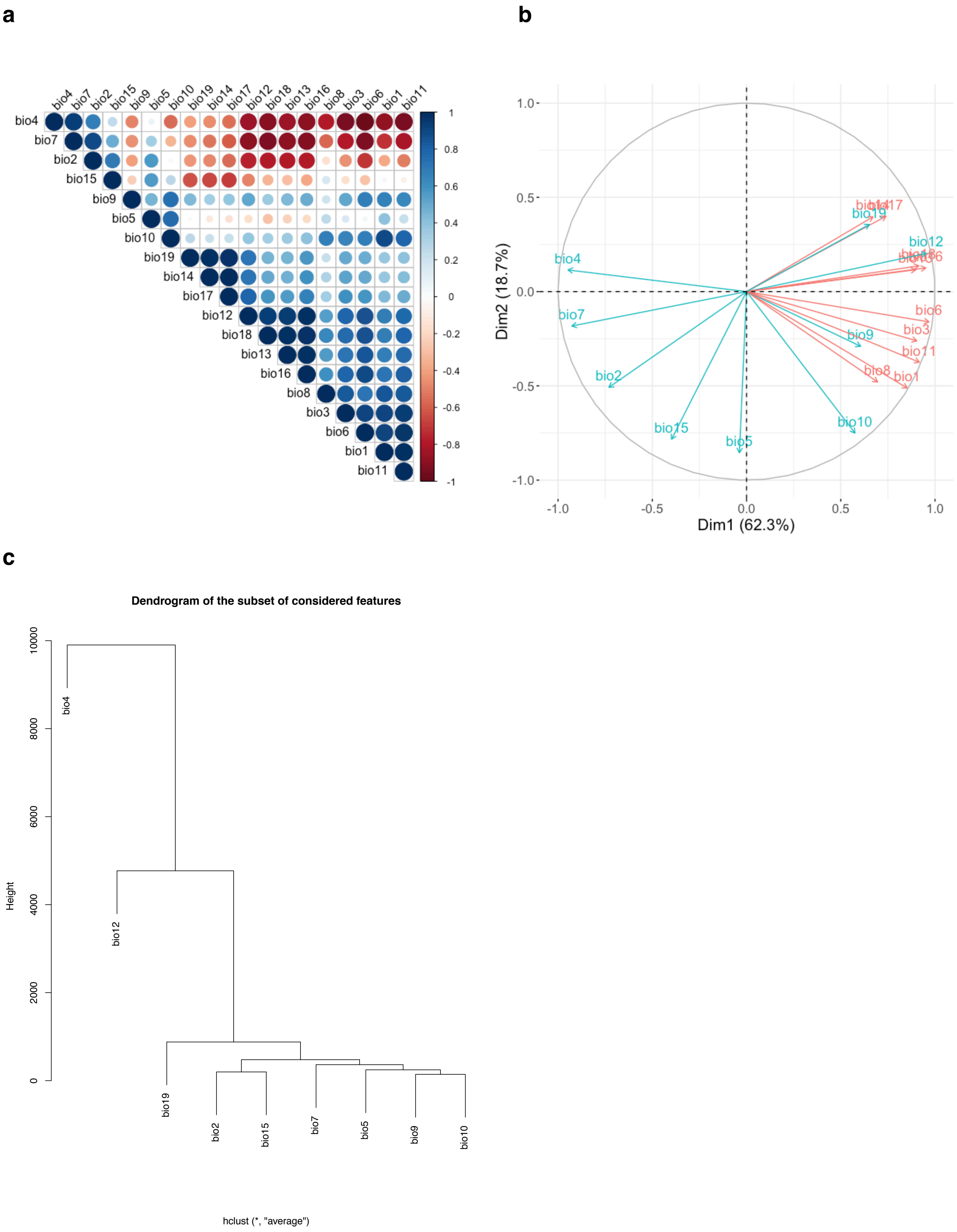
a
b
c
Dim1 (62.3%)
Dim2 (18.7%)
Dendrogram of the subset of considered features
Height
hclust (*, "average")
bio4
bio7
bio2
bio15
bio9
bio5
bio10
bio19
bio14
bio17
bio12
bio18
bio13
bio16
bio8
bio3
bio6
bio1
bio11

Supplementary Figure 16. Pairwise Pearson's correlations (a) and principal component analysis (b) between environmental variables from eight populations

(a) Matrix shows Pearson's correlation between bioclimatic variables determined from GPS coordinates for eight populations. Circle sizes indicate correlation intensity and colours correspond to correlation direction. Variables are clustered according to the correlation they entertain.

(b) Correlation circle shows bioclimatic variables coordinates on first two principal components. Red variables were considered as highly correlated and not considered further for gradient forest analysis.

(c) Dendrogram shows the nested correlation structure of remaining variables. To investigate whether the remaining correlation between variables was biasing our random forest framework, we implemented a hierarchical average clustering on the remaining variables. This approach identified 5 main clusters composed of BIO4, BIO12, BIO19, BIO2 and BIO15, and a last cluster containing BIO7, BIO5, BIO9 and BIO10. We ran a random forest analysis on the subset of variables from these clusters (BIO4, BIO12, BIO19, BIO2 and BIO7) and found BIO12 and BIO7 as the most important features. The same conclusion was found with BIO15 instead of BIO2. Our analysis identifying the importance of BIO12 and BIO7 thus seems robust to the details of variable selection.

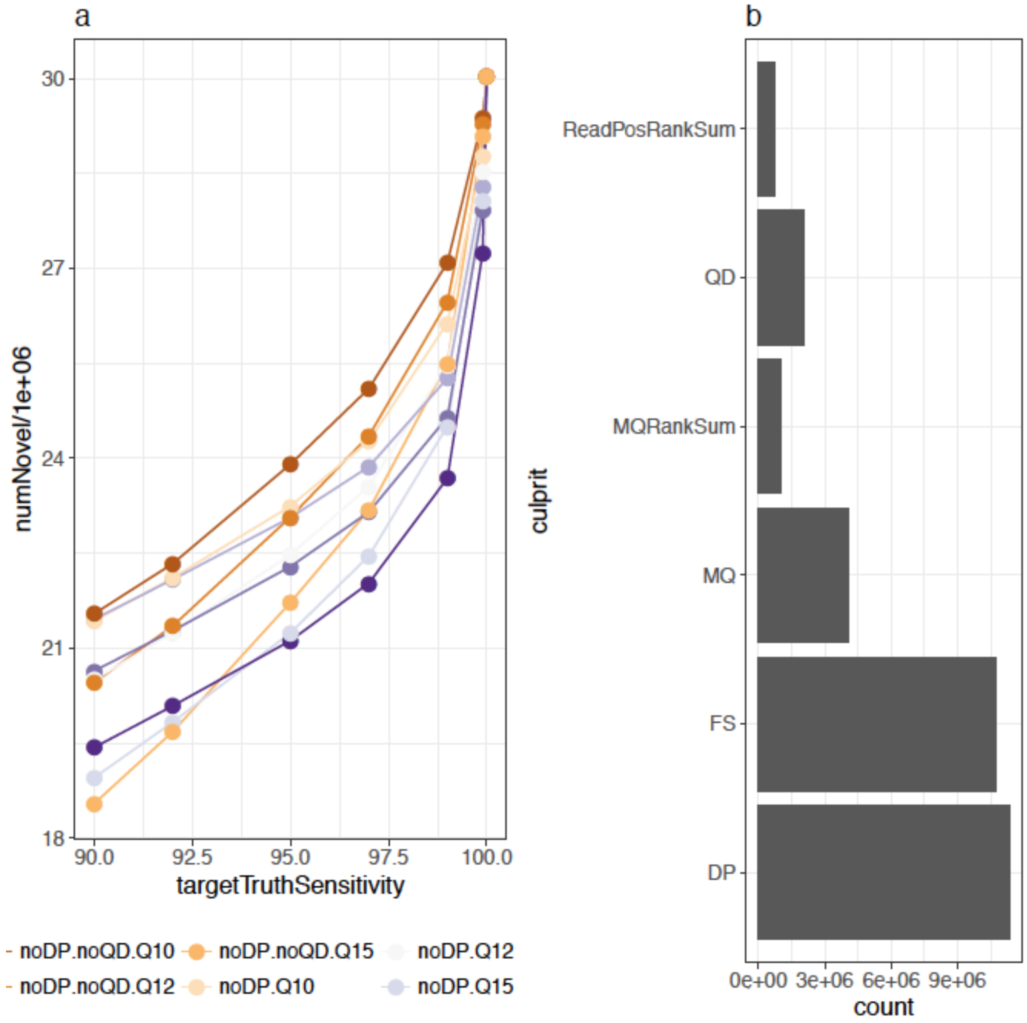

Supplementary Figure 17. Variant Quality Score Recalibration (VSQR) summary statistics

Supplementary Methods

Creation of a reference "truth" SNP database

The reference "truth" SNP database was generated from the intersection of variants called from samples with at least a mean of 10× coverage (n = 13) using three independent SNP callers: (i) samtools mpileup (-q20 –Q20 –C50 -uD), (ii) Freebayes[8] v.9.9.2-13-gad718f9-dirty (--min-mapping-quality 20 --min-alternate-count 5 --no-indels --min-alternate-qsum 40 --pvar 0.0001 --use-mapping-quality --posterior-integration-limits 1,3 --genotype-variant-threshold 4 --use-mapping-quality --site-selection-max-iterations 3 --genotyping-max-iterations 25 --max-complex-gap 3), and (iii) GATK HaplotypeCaller followed by hard filtering (--QD<2, --DP>10000, --FS>60, --MQ<40, --MQRS <-12.5, --RPRS<-8). The three variant call sets were merged (GATK CombineVariants with --genotypeMergeOptions UNIQUIFY), resulting in an intersecting set of 794,606 SNPs (extracted with GATK SelectVariants). The GATK VariantRecalibrator model was trained with this reference SNP database with a 90% prior likelihood, before being subsequently applied to the raw set of SNPs (n = 30,040,159). The estimation was run for several truth sensitivity threshold values ranging from 90 to 99.9%. After visual inspection of the additional number of SNPs by using sensitivity tranche curves (Supplementary Fig. 17a), a 97% sensitivity threshold was applied to the raw SNP set with GATK ApplyRecalibration, resulting in a total set of 23,868,644 SNPs spanning the five autosomes. Variant depth of coverage (DP) and strand bias (FS) were the main drivers of SNP removal (Supplementary Fig. 17b).

Effective population size estimation with MSMC2

The effective population size (*Ne*) trajectory through time, and the cross-divergence time between populations, were estimated using MSMC2[9,10]. This approach uses patterns of heterozygosity along the genome to identify past recombination events modelled as Markov processes[11].

Mutation density along the sequence mirrors either recent (long tract of limited diversity) or older (enrichment in heterozygosity over short distances) events. According to coalescent theory[12], at any given time, the amount of recombination is proportional to *Ne*.

Input files were created following MSMC recommendations and available msmc-tools (https://github.com/stschiff/msmc-tools). Briefly, the reference fasta sequence was masked with SNPable (http://lh3lh3.users.sourceforge.net/snpable.shtml) to extract regions of unambiguous read mapping in chromosome-specific bed files (using the available msmc_create_map_mask.py python script). Negative bed files indicating regions with sufficient coverage at the individual level were created from samples bam files with the bamCaller.py script and filter out sites with coverage below genome-wide average depth. Finally, MSMC2 input files were created for each chromosome with the generate_multihetsep.py script and concatenated into a single input file. For each isolate, estimates were averaged across five runs, leaving one chromosome out at a time for cross-validation, using rho/mu parameter value of 6.22 (average recombination rate of 1.68 cM/Mbp[13] and considering a mutation rate similar to that of *C. elegans* mutation rate, 2.7 x 10^{-9} per site per generation[14]). MSMC2 times and coalescent rates were scaled to real time and population sizes by assuming the same mutation rate[14], a balanced sex-ratio[15] and an inferred generation interval of 40 days (the sum of 10 days to reach mature free-living infective larvae from the egg stage, and a 30-day prepatent period for fully mature egg-laying females)[16].

Modeling population demographics and divergence dating

Migratory scenarios between populations were determined using the forward simulation framework implemented in δaδi[17]. For each model, four rounds of forward simulations were run with 10, 20, 30 and 40 replicates respectively using published python scripts[18] (https://github.com/dportik/dadi_pipeline). Model Akaike Information Criterion (AIC) were

compared for ranking scenarios, from which the lower the score, the more likely the outcome. We first compared a divergence scenario without migration against models including symmetrical and asymmetrical gene flow before isolation. In case migration was the most likely, more complex models (involving split with ancestral (a)symmetrical gene flow, with or without population size change, or models involving secondary contact with/without gene flow and population size change) were tested. However, initial exploration indicated a likely lack of power in our design to accurately estimate parameters of more complex demographic models than the split and isolation model. Nevertheless, these models still provide the most likely scenario and their output have been listed in supplementary Data 2.

Parameters were scaled to real time using same parameters as for MSMC2 inference. Standard deviations of timing estimates for the simple split and isolation models were obtained using the Godambe Information Matrix[19] applied to 100 simulated site frequency spectra produced with the *ms* software[20] under the most likely demographic model.

Phylogenetic analysis of mitochondrial coding sequences

Additional support to the estimates from the nuclear genome were obtained from a phylogenetic analysis of mitochondrial coding sequences using BEASTv1.10[21]. Mitochondrial coding sequences were extracted from the consensus sequence of every individual, concatenated per individual, and aligned using Musclev3.8.31[22]. A Bayesian skyline model[21] was used, with a HKY substitution model and a strict clock model, as other modalities yielded weak effective sample size (ESS) and unstable parameter values. Clock rate was set to *C. elegans* mitochondrial mutation rate, i.e. 1×10^{-7} per site per generation[23], as variation in sampling date was not sufficient to estimate molecular rate. Parameters showed sufficient sampling (effective size above 200) after 50,000,000 iterations and a burn-in of the first 20 million steps. Node ages were scaled to

years assuming a generation interval of 40 days. A maximum clade credibility tree was generated with TreeAnnotator v1.10.1 (http://beast.community/treeannotator).

Supplementary Notes

Supplementary Note 1. Evaluation of genotype analysis frameworks in face of low coverage samples

Our sequencing effort achieved a mean coverage of 3x across 223 individuals, thus hampering stringent filtering on SNP genotypes. Two options were implemented to deal with this matter: (i) the Variant Quality Score Recalibration of the GATK[24] software, or (ii) the probabilistic framework implemented in the ANGSD[2] software that relies on genotype likelihoods (GLs). VQSR SNP calls were considered for analyses that were not available in ANGSD, or when bias was identified in ANGSD output (average pair-wise F_{ST} between populations).

To compare the impact of coverage between the two approaches, we used a subset of 43 individuals that underwent an additional round of sequencing and applied the analyses on this dataset before (mean coverage of 2.47x [1.55 – 4.48]; hereafter referred to as “Low coverage set”) and after (mean coverage of 8x [3.35 – 15.24]; hereafter referred to as “High coverage set”) re-sequencing (Supplementary Figure 18).

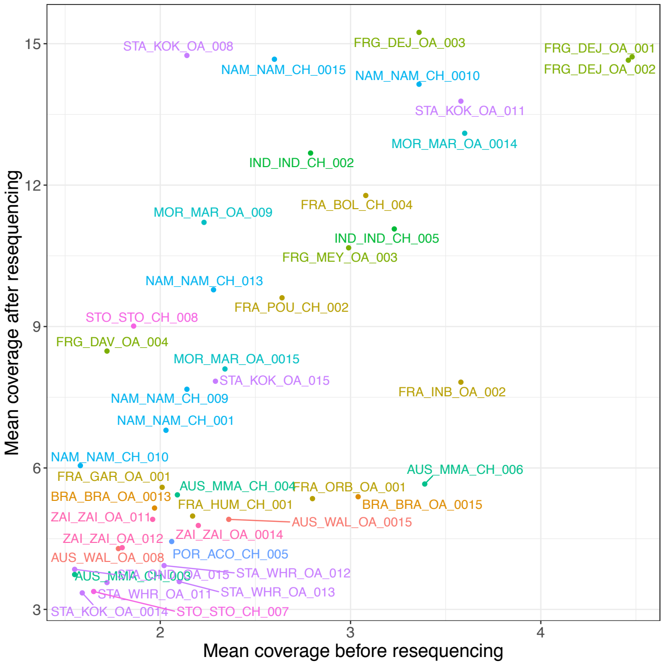

Supplementary Figure 18. Coverage improvement for a subset of 43 individual *Haemonchus contortus* males

Mean coverage of 43 samples (coloured by isolate) after resequencing is plotted against their respective mean depth of coverage before resequencing.

Following this approach, we compared nucleotide diversity estimates and Tajima's *D* estimates in three populations exhibiting highest number of individuals with 5x mean coverage and more, i.e. Namibia (NAM), France (FRA.1) and Guadeloupe (GUA). Results (Supplementary Figure 18) demonstrated that nucleotide diversity estimates (shown for Chromosome I) were significantly biased downward in the low coverage set (-0.135%, $P < 10^{-4}$), while Tajima's *D* statistic was higher in this set (+0.028, $P < 10^{-4}$). However, distribution pattern along the considered chromosome was not altered.

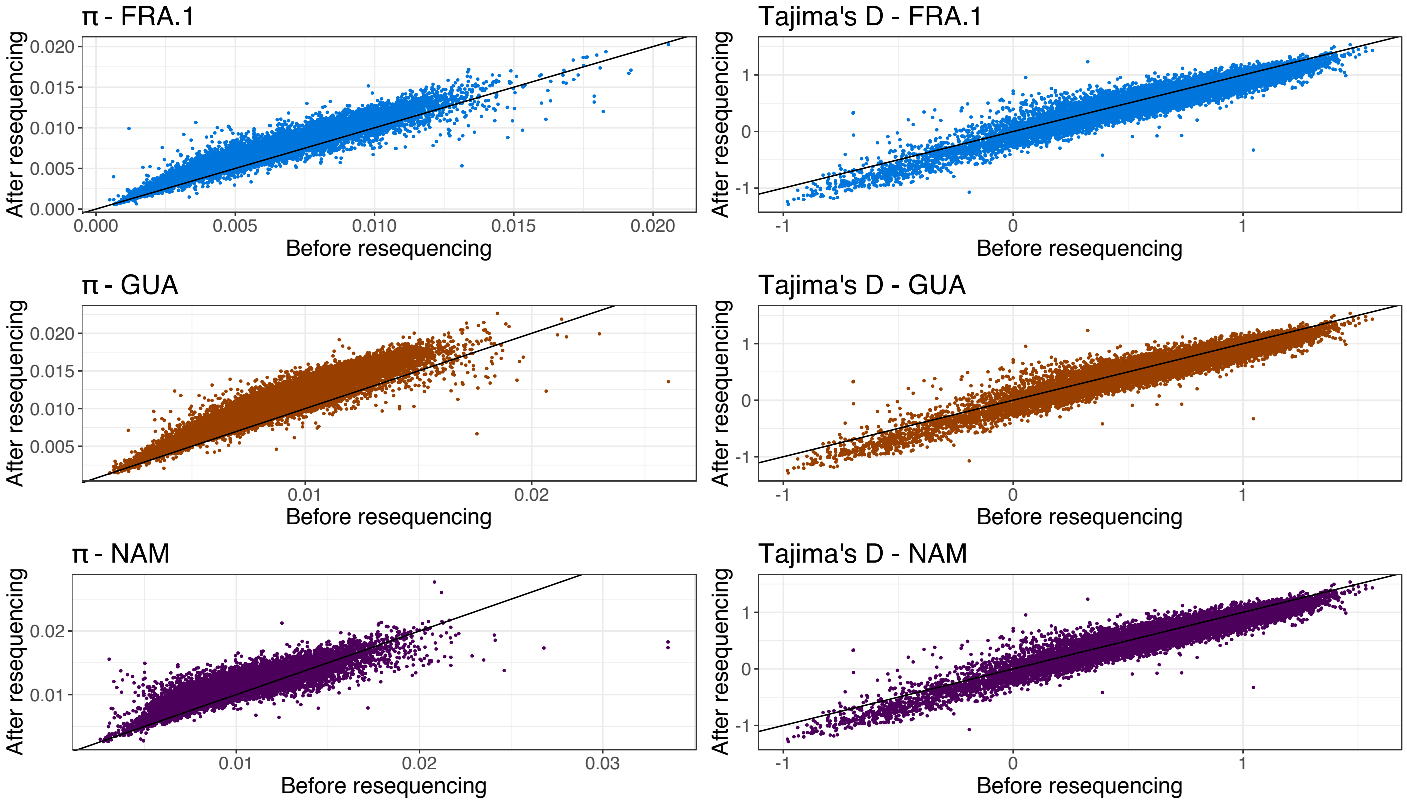

Supplementary Figure 19. The relationship between sample coverage and population diversity estimates

Nucleotide diversity (left panels) or Tajima's *D* (right panels) estimates computed from the same samples before (x-axis) or after (y-axis) resequencing are plotted for the French, Guadeloupian and Namibian populations (coloured in blue, brown and purple respectively).

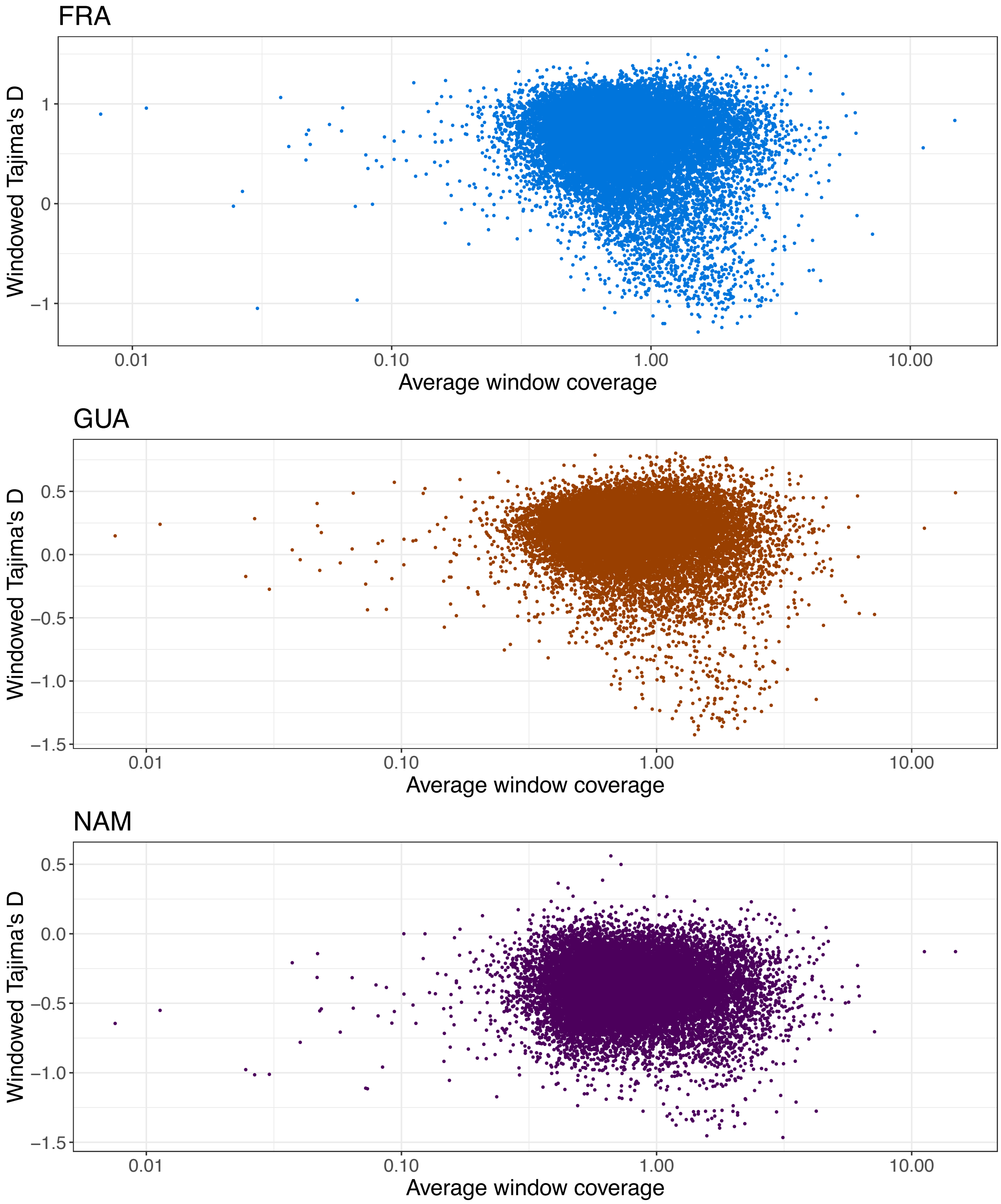

Supplementary Figure 20. The relationship between Tajima's *D* estimates and coverage

Tajima's D values, estimated under the probabilistic framework of ANGSD over 10 Kbp windows, have been plotted as a function of local depth of coverage within respective windows for three populations of interest (FRA: France mainland; NAM: Namibia; GUA: Guadeloupe). This figure indicates weak correlation between Tajima's D values and coverage (Pearson's r = -0.17, -0. 13 and -0.04 for FRA, GUA and NAM respectively).

Pairwise F_{ST} estimates from ANGSD were also correlated with population cross-coverage (Pearson's $r_{(136)} = 0.54$, $P<10^{-4}$), suggestive of a bias. This bias could be overcome when considering VQSR SNP calls and using the maximal value binned by 10% minor allele frequency (supplementary figure 21). In this case, correlation was not significant (Pearson's $r_{(136)} = -0.05$, $P = 0.55$).

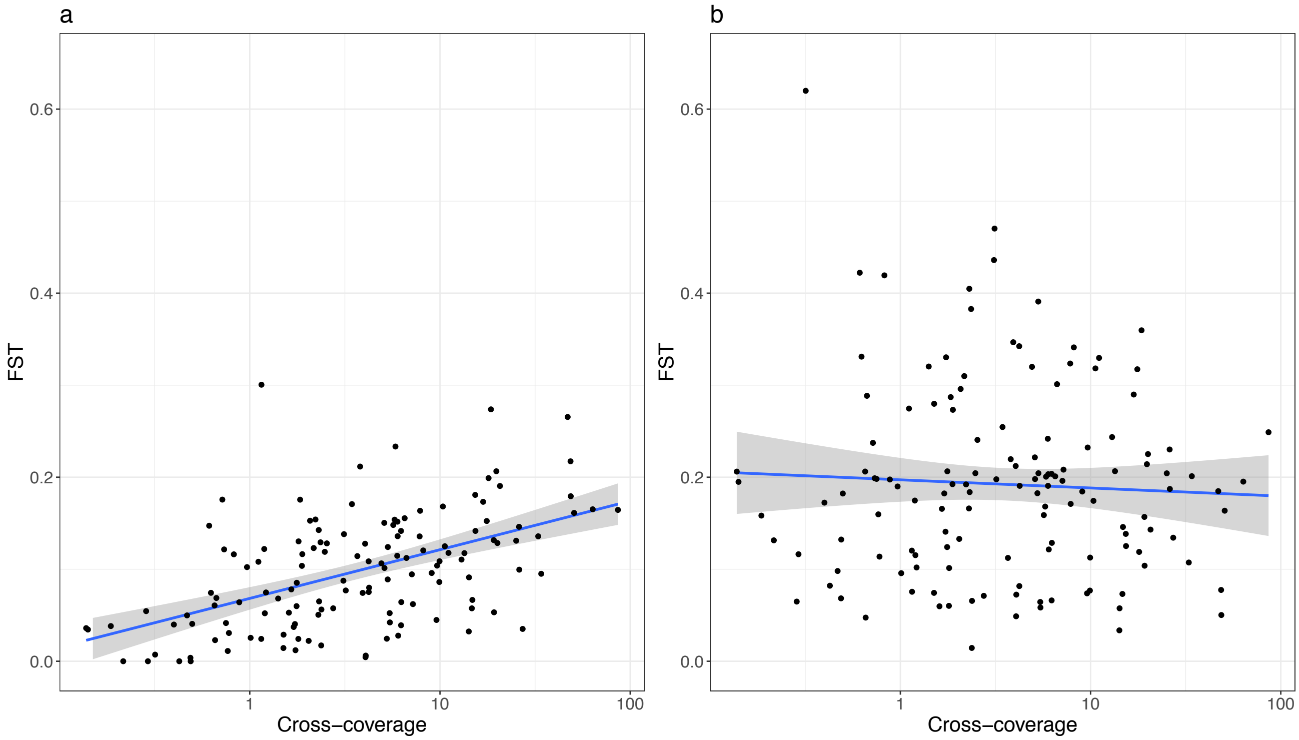

Supplementary Figure 21. The relationship between pairwise F_{ST} estimates and coverage according to the considered framework

F_{ST} values, estimated under the probabilistic framework of ANGSD (a) or using the maximal value of F_{ST} binned by minor allele frequency (b) have been plotted as a function of population cross-coverage. This latter estimate was retained in our analyses as reported elsewhere[25].

Pairwise divergence between individuals was also affected by coverage, as Hamming distance estimates based on VQSR SNP calls increased as cross-coverage increased (Pearson' $r_{(49729)}$ = 0.31, $P<10^{-4}$; supplementary Figure 22a). To account for this, the sole individuals exhibiting a mean depth of coverage higher than 2.5x were considered resulting in bias removal (Pearson' $r_{(8281)}$ = 0.02, P = 0.09; supplementary Figure 22b).

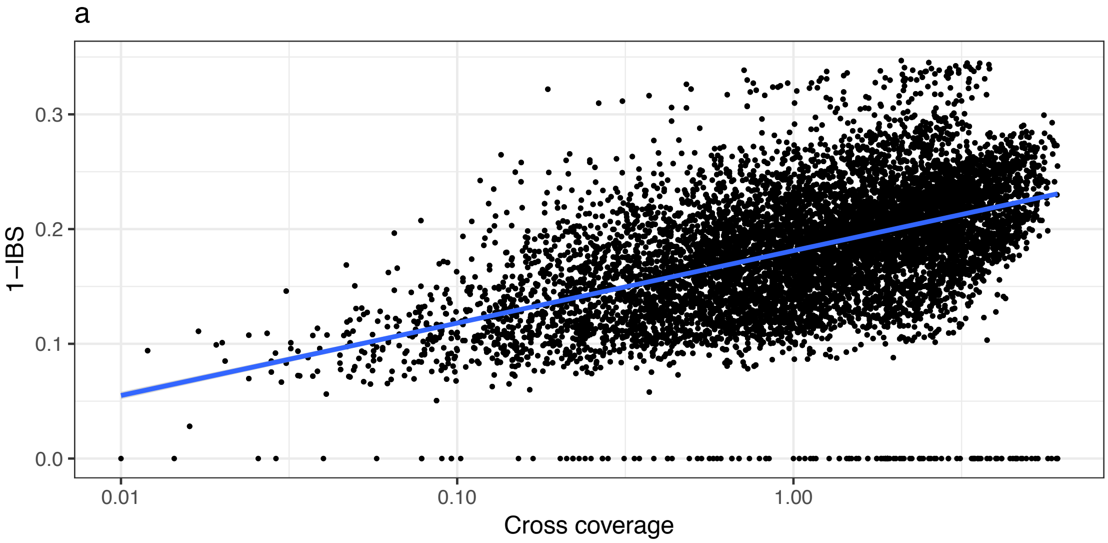

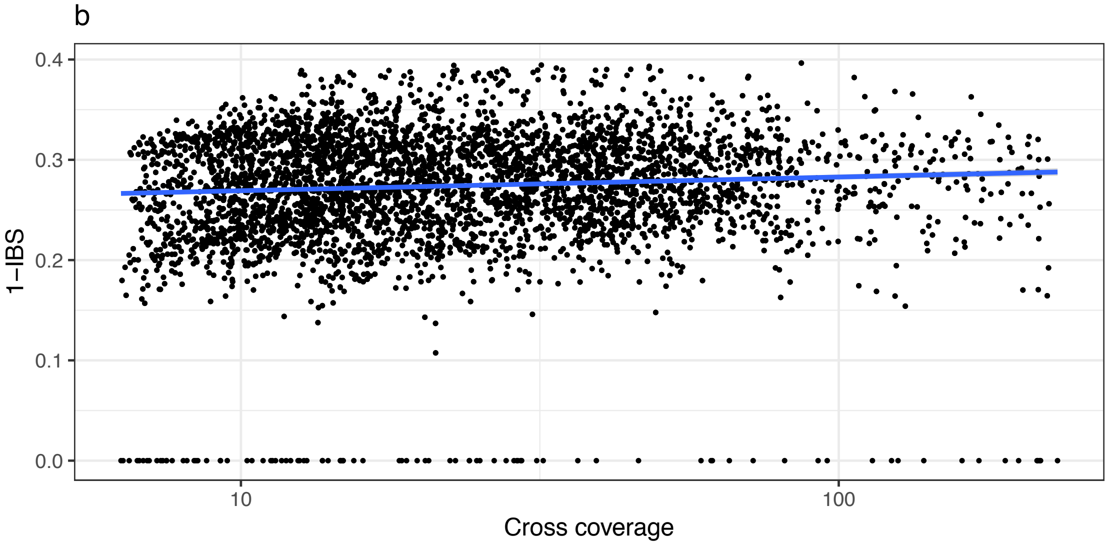

Supplementary Figure 22. The relationship between Hamming's distance and coverage

Both frameworks also yielded similar population clustering. ANGSD provided more distinct clusters, especially for populations with the least coverage like Benin and the 4th French population (FRA.4). The only discrepancy was the clustering of Indonesian population among Guadeloupian samples when considering VQSR SNP calls (supplementary figure 23). The low proportion of variance explained by the first two components with ANGSD (figure 1b) remains unexplained. The first two components of a PCA on VQSR SNP explained 7.96% and 5.86% respectively.

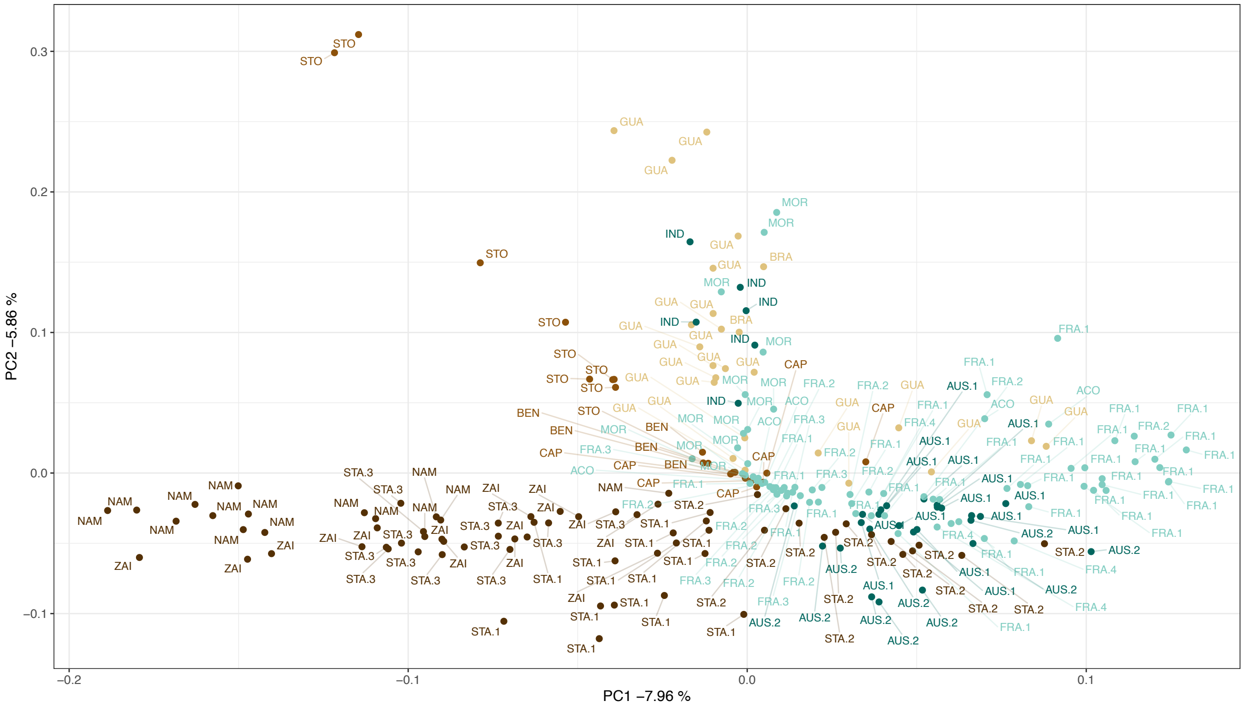

Supplementary Figure 23. Principal component analysis based on nuclear VQSR SNP calls across 223 individuals

Each dot stands for an individual and colours indicate the geographical origin of the sample (sand: America; light green: Mediterranean area; dark green: Oceania; brown: Western Africa; dark brown: South-African area)

Admixture analysis was robust to coverage (supplementary figure 24) as demonstrated by an analysis on the 43 samples that underwent re-sequencing. Population ancestry pattern inferred from re-sequenced samples (mean coverage of 8x [3.35 – 15.24]; right column) remained similar to that observed before re-sequencing (mean coverage of 2.47x [1.55 – 4.48]; left column).

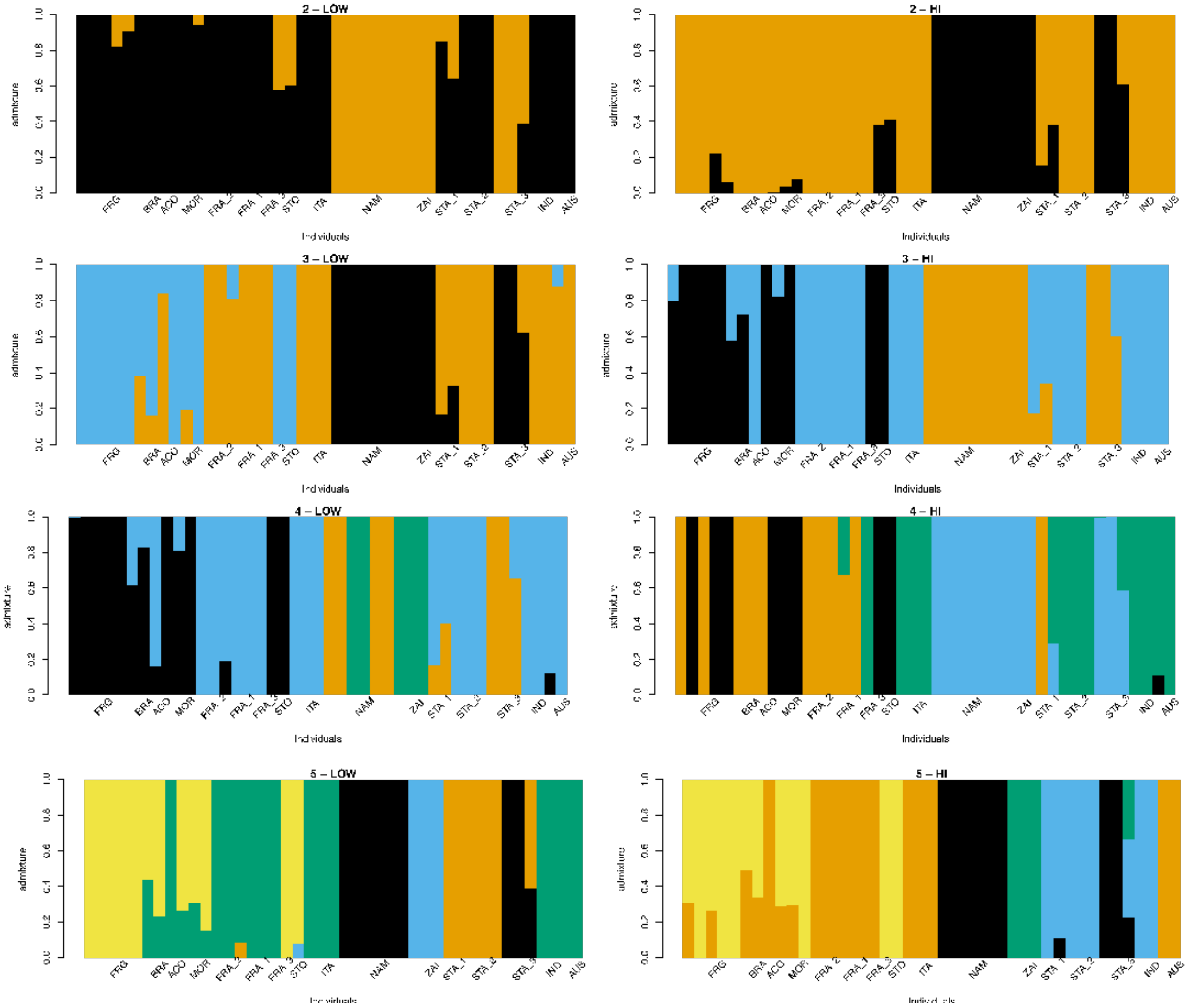

Supplementary Figure 24. Admixture analysis run for chromosome II on the same set of 43 individuals before (left) or after (right) resequencing for K ranging from 2 to 5

Supplementary Note 2. Beagle imputation accuracy

The topology weighting analysis implemented with TWISST and the cross-coalescent time estimation both relied on Beagle[26] imputed genotypes. To evaluate how imputation accuracy would perform on low coverage samples, Beagle was run for chromosome I on the subset of 43 re-sequenced samples, using the genotypes called on low coverage BAM files (before re-sequencing). Discordance at the individual and site levels between imputed genotypes and genotype called using data after resequencing were subsequently computed with vcftools[27] v0.1.15 using the --diff-site-discordance and --diff-indv-discordance options. At the individual level, the mean discordance was 7.2% [3.4% – 18.97%]. Discordance level was consistent throughout the chromosome (supplementary figure 25a) and two third of the sites displayed less than 10% (supplementary figure 25b) discordance between imputed genotypes and genotype called from high coverage BAM files. Discordance displayed was slightly increased for samples with higher coverage before resequencing (Supplementary Figure 25c).

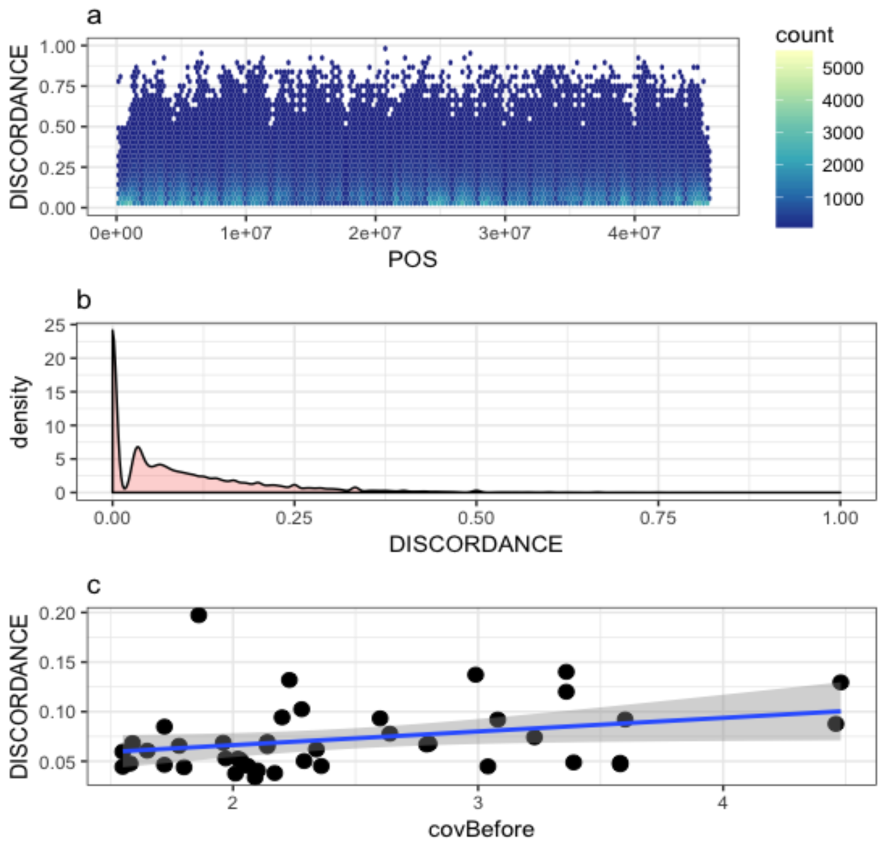

Supplementary Figure 25. Genotype discordance after Beagle imputation

(a) the density distribution of discordance (ranging between 0 and 100%) for SNP sites (binned into hexagons) along chromosome I between imputed genotypes and called genotype of the same sample with high coverage.

(b) the density distribution of discordance values at the SNP site level, highlighting that a majority of sites show less than 10% discordance between imputed and called genotypes with high coverage data.

(c) the relationship between individual coverage before resequencing and the average discordance at the SNP level for chromosome I.

Supplementary references

1 Oosthuizen, W. T. & Erasmus, J. B. Efficacy of moxidectin against a strain of Haemonchus contortus resistant to ivermectin, a benzimidazole and a salicylanilide. *J S Afr Vet Assoc* **64**, 9-12 (1993).
2 Korneliussen, T. S., Albrechtsen, A. & Nielsen, R. ANGSD: Analysis of Next Generation Sequencing Data. *BMC Bioinformatics* **15**, 356 (2014).
3 Small, S. T. *et al.* Population genomics of the filarial nematode parasite Wuchereria bancrofti from mosquitoes. *Mol Ecol* **25**, 1465-1477 (2016).
4 Choi, Y. J. *et al.* Genomic diversity in Onchocerca volvulus and its Wolbachia endosymbiont. *Nat Microbiol* **2**, 16207 (2016).
5 Langley, C. H. *et al.* Genomic variation in natural populations of Drosophila melanogaster. *Genetics* **192**, 533-598 (2012).
6 Chang, C. C. *et al.* Second-generation PLINK: rising to the challenge of larger and richer datasets. *Gigascience* **4**, 7 (2015).
7 Skotte, L., Korneliussen, T. S. & Albrechtsen, A. Estimating individual admixture proportions from next generation sequencing data. *Genetics* **195**, 693-702 (2013).
8 Garrison, E. & Marth, G. Haplotype-based variant detection from short-read sequencing. *arXiv preprint* **arXiv:1207.3907 [q-bio.GN]** (2012).
9 Schiffels, S. & Durbin, R. Inferring human population size and separation history from multiple genome sequences. *Nat Genet* **46**, 919-925 (2014).
10 Malaspinas, A. S. *et al.* A genomic history of Aboriginal Australia. *Nature* **538**, 207-214 (2016).
11 McVean, G. A. & Cardin, N. J. Approximating the coalescent with recombination. *Philos Trans R Soc Lond B Biol Sci* **360**, 1387-1393 (2005).
12 Kingman, J. F. C. The coalescent. *Stochastic Processes and their Applications* **13**, 235-248 (1982).
13 Doyle, S. R. *et al.* A Genome Resequencing-Based Genetic Map Reveals the Recombination Landscape of an Outbred Parasitic Nematode in the Presence of Polyploidy and Polyandry. *Genome Biol Evol* **10**, 396-409 (2018).
14 Denver, D. R. *et al.* A genome-wide view of Caenorhabditis elegans base-substitution mutation processes. *Proc Natl Acad Sci U S A* **106**, 16310-16314 (2009).
15 Saccareau, M. *et al.* Meta-analysis of the parasitic phase traits of Haemonchus contortus infection in sheep. *Parasit Vectors* **10**, 201 (2017).
16 Emery, D. L., Hunt, P. W. & Le Jambre, L. F. Haemonchus contortus: the then and now, and where to from here? *Int J Parasitol* **46**, 755-769 (2016).
17 Gutenkunst, R. N., Hernandez, R. D., Williamson, S. H. & Bustamante, C. D. Inferring the joint demographic history of multiple populations from multidimensional SNP frequency data. *PLoS Genet* **5**, e1000695 (2009).
18 Portik, D. M. *et al.* Evaluating mechanisms of diversification in a Guineo-Congolian tropical forest frog using demographic model selection. *Mol Ecol* **26**, 5245-5263 (2017).
19 Coffman, A. J., Hsieh, P. H., Gravel, S. & Gutenkunst, R. N. Computationally Efficient Composite Likelihood Statistics for Demographic Inference. *Mol Biol Evol* **33**, 591-593 (2016).

20 Hudson, R. R. Generating samples under a Wright-Fisher neutral model of genetic variation. *Bioinformatics* **18**, 337-338 (2002).

21 Drummond, A. J., Rambaut, A., Shapiro, B. & Pybus, O. G. Bayesian coalescent inference of past population dynamics from molecular sequences. *Mol Biol Evol* **22**, 1185-1192 (2005).

22 Edgar, R. C. MUSCLE: multiple sequence alignment with high accuracy and high throughput. *Nucleic Acids Res* **32**, 1792-1797 (2004).

23 Konrad, A. *et al.* Mitochondrial Mutation Rate, Spectrum and Heteroplasmy in Caenorhabditis elegans Spontaneous Mutation Accumulation Lines of Differing Population Size. *Mol Biol Evol* **34**, 1319-1334 (2017).

24 McKenna, A. *et al.* The Genome Analysis Toolkit: a MapReduce framework for analyzing next-generation DNA sequencing data. *Genome Res* **20**, 1297-1303 (2010).

25 Svardal, H. *et al.* Ancient hybridization and strong adaptation to viruses across African vervet monkey populations. *Nat Genet* **49**, 1705-1713 (2017).

26 Browning, B. L. & Browning, S. R. A unified approach to genotype imputation and haplotype-phase inference for large data sets of trios and unrelated individuals. *Am J Hum Genet* **84**, 210-223 (2009).

27 Danecek, P. *et al.* The variant call format and VCFtools. *Bioinformatics* **27**, 2156-2158 (2011).
