# Supplementary material for: The global diversity of Haemonchus contortus is shaped by human intervention and climate
Source: Nat Commun. 2019 Oct 22;10:4811. doi: 10.1038/s41467-019-12695-4 (PMC6805936; doi:10.1038/s41467-019-12695-4)
Supplement: Supplementary file 3 — Reporting Summary [file 41467_2019_12695_MOESM3_ESM.pdf]

nature research

Corresponding author(s): Guillaume SALLE

Last updated by author(s): Aug 30, 2019

Reporting Summary

Nature Research wishes to improve the reproducibility of the work that we publish. This form provides structure for consistency and transparency in reporting. For further information on Nature Research policies, see Authors & Referees and the Editorial Policy Checklist .

Statistics

For all statistical analyses, confirm that the following items are present in the figure legend, table legend, main text, or Methods section.

n/a	Confirmed	
☐	☒	The exact sample size (*n*) for each experimental group/condition, given as a discrete number and unit of measurement
☒	☐	A statement on whether measurements were taken from distinct samples or whether the same sample was measured repeatedly
☐	☒	The statistical test(s) used AND whether they are one- or two-sided<br>*Only common tests should be described solely by name; describe more complex techniques in the Methods section.*
☐	☒	A description of all covariates tested
☐	☒	A description of any assumptions or corrections, such as tests of normality and adjustment for multiple comparisons
☐	☒	A full description of the statistical parameters including central tendency (e.g. means) or other basic estimates (e.g. regression coefficient) AND variation (e.g. standard deviation) or associated estimates of uncertainty (e.g. confidence intervals)
☐	☒	For null hypothesis testing, the test statistic (e.g. *F*, *t*, *r*) with confidence intervals, effect sizes, degrees of freedom and *P* value noted<br>*Give P values as exact values whenever suitable.*
☐	☒	For Bayesian analysis, information on the choice of priors and Markov chain Monte Carlo settings
☐	☒	For hierarchical and complex designs, identification of the appropriate level for tests and full reporting of outcomes
☐	☒	Estimates of effect sizes (e.g. Cohen's *d*, Pearson's *r*), indicating how they were calculated

Our web collection on statistics for biologists contains articles on many of the points above.

Software and code

Policy information about availability of computer code

Data collection	No software was needed for data collection.
Data analysis	The raw sequencing and genotype data were analysed using publicly available softwares as described in main text. Outputs of these analyses were used for statistical analyses and figure drawing with a custom R script available at: https://github.com/guiSalle/Haemonchus_diversity. R and packages versions have been appended at the end of this R script. Additional custom scripts used to generate some of these data are also available under the same repository.

For manuscripts utilizing custom algorithms or software that are central to the research but not yet described in published literature, software must be made available to editors/reviewers. We strongly encourage code deposition in a community repository (e.g. GitHub). See the Nature Research guidelines for submitting code & software for further information.

Data

Policy information about availability of data

All manuscripts must include a data availability statement. This statement should provide the following information, where applicable:

- Accession codes, unique identifiers, or web links for publicly available datasets
- A list of figures that have associated raw data
- A description of any restrictions on data availability

Raw sequencing data are archived under the ENA study accession PRJEB9837. Sequencing data were analysed with publicly available softwares as listed in main text. Output of these softwares were analysed using an R script available at: https://github.com/guiSalle/Haemonchus_diversity. Data for figure reproduction have also been made available under this repository. Reference assembly used in this project is available at: ftp://ngs.sanger.ac.uk/production/pathogens/Haemonchus_contortus.

Field-specific reporting

Please select the one below that is the best fit for your research. If you are not sure, read the appropriate sections before making your selection.

☐ Life sciences ☐ Behavioural & social sciences ☒ Ecological, evolutionary & environmental sciences

For a reference copy of the document with all sections, see nature.com/documents/nr-reporting-summary-flat.pdf

Ecological, evolutionary & environmental sciences study design

All studies must disclose on these points even when the disclosure is negative.

Study description	Our study describes the population structure of 19 Haemonchus contortus (sheep worm) populations.
Research sample	The sampling regime was motivated to delineate the contribution of major evolutionary forces, i.e. migration and selection (drug and climate). Because migration was likely to match human history, isolates from western African countries and southern America were selected to address the contribution of slave trade history to the structuring of H. contortus populations; isolates from former colonies of the British Empire (South-Africa, Australia) were sampled to establish the connectivity between worm populations from Europe and these countries. Ivermectin-resistant isolates (AUS.1, STA.1, STA.3) were also retained to evaluate how anthelmintics had shaped H. contortus genomic variability. Isolates were selected based on available material to ensure minimal sample size (n = 9) per isolate and proper allele frequency estimation. Following these criteria, 19 isolates from 12 countries were available.
Sampling strategy	Worms were collected from sheep or goat in abattoir or during necropsy, before being stored in alcohol and deep-frozen in liquid nitrogen.
Data collection	Metadata associated with each worm were collected by the INRA biobank. Genomic data were generated by the Wellcome Sanger Institute sequencing team.
Timing and spatial scale	Worms were added to the biobank between 1995 and 2011, following research needs, collaborations or field work. Isolates range spans 12 countries from America, Europe, Africa, Oceania and Asia.
Data exclusions	After an initial sequencing round, two samples appeared to be contaminated and were discarded from further analyses. This step and the whole description of data processing can be found under the materials and methods section.
Reproducibility	Sequencing data were analysed with publicly available softwares as listed in main text. Output of these softwares were analysed using an R script available at: https://github.com/guiSalle/Haemonchus_diversity. Data for figure reproduction have also been made available under this repository.
Randomization	Because this study relies on a worm biobank from the field, no particular groups could be made. We ensured that samples from various countries/continents or with different drug susceptibility were multiplexed on the same sequencing batch to avoid technical biases.
Blinding	Blinding was not relevant to this study as we needed to know the origin or drug susceptibility status of each individual. Nevertheless, attempts to localize genes underpinning major biological function were made without prior knowledge about gene positions on the genome.

Did the study involve field work? ☐ Yes ☒ No

Reporting for specific materials, systems and methods

We require information from authors about some types of materials, experimental systems and methods used in many studies. Here, indicate whether each material, system or method listed is relevant to your study. If you are not sure if a list item applies to your research, read the appropriate section before selecting a response.

Materials & experimental systems

n/a	Involved in the study	
☒	☐	Antibodies
☒	☐	Eukaryotic cell lines
☒	☐	Palaeontology
☐	☒	Animals and other organisms
☒	☐	Human research participants
☒	☐	Clinical data

Methods

n/a	Involved in the study	
☒	☐	ChIP-seq
☒	☐	Flow cytometry
☒	☐	MRI-based neuroimaging

Animals and other organisms

Policy information about studies involving animals; ARRIVE guidelines recommended for reporting animal research

Laboratory animals	The study did not involve laboratory animals.
Wild animals	The study did not involve wild animals.
Field-collected samples	Worm samples were stored deep frozen in liquid nitrogen.
Ethics oversight	No ethical approval was required as our work focuses on an invertebrate species.

Note that full information on the approval of the study protocol must also be provided in the manuscript.
