# Supplementary material for: The global diversity of Haemonchus contortus is shaped by human intervention and climate
Source: Nat Commun. 2019 Oct 22;10:4811. doi: 10.1038/s41467-019-12695-4 (PMC6805936; doi:10.1038/s41467-019-12695-4)
Supplement: Supplementary file 4 — Description of Additional Supplementary Files [file 41467_2019_12695_MOESM4_ESM.pdf]

Description of Additional Supplementary Files

File Name: Supplementary Data 1
Description: **Metadata describing the provenance and sequencing of individual Haemonchus contortus samples**
Unless stated otherwise (France, Guadeloupe, South-Africa and Australia), isolates were sampled from a single farm within each country.

File Name: Supplementary Data 2
Description: **Maximum-likelihood parameter estimates obtained from the joint demographic inference analysis**
For every population, unscaled parameters from forward genetic simulations run in δaδi are listed. Initial exploration of the fit of more complex scenarios than a simple split and isolation model suggested there was little power in our data to estimate parameters accurately under these models. Nevertheless, outputs from these models are shown as an indication of the most likely demographic scenario between corresponding populations. For each model and pair of populations (population1-population2), estimation round and replicate are provided along with considered genomic segment length (L), model loglikelihood, Akaike Information Criterion (AIC). Nu1a and nu2a correspond to population sizes after first event and nu1b and nu2b indicate population size following secondary event if any. In case of asymmetrical migration between populations, two migration rates are provided (m12, m21 for migration from population1 to population2 and from population2 to population 1 respectively). Unscaled timings of events appear as T1 (time of split), T2 (time since secondary event) and T3 (time since third event if any) and corresponding years under the common era (CE) have been listed as Year of split, Year of secondary event, Year of isolation respectively. Standard deviations of parameters estimates are indicated in brackets for simple “split and isolation” models. Asym: asymmetrical; Sym: symmetrical.

File Name: Supplementary Data 3
Description: **Positional candidate genes overlapping significant XP-CLR selection score**

File Name: Supplementary Data 4
Description: **Significant GO term enrichment from genes under significant diversifying selection in at least one of the populations pairwise comparison**

File Name: Supplementary Data 5
Description: **Differentiated windows between populations from contrasting climatic conditions**
